# Nearfield observation of spin-orbit interactions at nanoscale using photoinduced force microscopy

**DOI:** 10.1126/sciadv.adp8460

**Published:** 2024-12-20

**Authors:** Yajuan Dong, Yu Wang, Dengji He, Tao Wang, Jinwei Zeng, Jian Wang

**Affiliations:** ^1^ Wuhan National Laboratory for Optoelectronics and School of Optical and Electronic Information, Huazhong University of Science and Technology, Wuhan 430074, Hubei, China.; ^2^ Optics Valley Laboratory, Wuhan 430074, Hubei, China.

## Abstract

Optical spin and orbital angular momenta are intrinsic characteristics of light determined by its polarization and spatial degrees of freedom, respectively. At the nanoscale, sharply focused structured light carries coupled spin-orbital angular momenta with complex 3D nearfield structures, crucial for manipulating multidimensional information of light in nanophotonics. However, characterizing these interactions faces challenges with conventional farfield–based methods, which typically lack the essential accuracy and resolution to interrogate the structured nearfield with high fidelity. To address this challenge, we experimentally observe spin-orbit interactions at the nanoscale using photoinduced force microscopy. Such interactions are enabled through sharply focused circularly polarized optical vortices, which are then mapped by nearfield optical forces in high resolution. Because the optical forces can reveal both longitudinal and transverse nearfield structures with high fidelity, the spin-orbit interactions are eventually evaluated quantitatively at the nearfield, as an important inspiration to use the coupled momenta in dense optical nano-device systems.

## Teaser

3D complex spin-orbital conversion in super-resolution is interrogated through nearfield optical force.

## INTRODUCTION

Light can have both spin angular momentum (SAM) and orbital angular momentum (OAM), which are associated with polarization and spatial degrees of freedom (DOFs), respectively. On one hand, the right- or left-handed circularly polarized (RCP or LCP) light beams carry SAM with ±ℏ per photon that corresponds to polarization helicity σ=±1 (from the view of the source), respectively (*1*); on the other hand, the structured light beams with lℏ per photon, where l is the topological charge and φ is the azimuthal angle (*2*, *3*).

Conventionally, the SAM and OAM are considered independent physical dimensions of light for the paraxial beam at the farfield. With the recent development of nanotechnologies, spin-orbit interactions (SOIs) can be enabled and observed under non-paraxiality and/or inhomogeneity conditions. It is a phenomenon that involves the mutual interaction and coupling of spin and orbital properties, governing the spatial DOFs of light, primarily manifested in the intensity distribution and propagation paths of light (*4*–*6*). Such SOIs play a vital role in a variety of light-matter interactions, such as the spin-Hall effect, spin-orbit Hall effect, spin-orbit conversion (SOC), and spin-controlled unidirectional excitation (*7*–*11*). Consequently, this has led to a variety of applications in modern optics, including optical mechanisms and manipulations, optical imaging and microscopy, and precision metrology, etc. (*12*–*15*).

Typical optical SOIs in non-paraxial systems and inhomogeneous conditions require special light manipulation at the nanoscale. This involves a precise subwavelength effect achieved through intrinsic coupling between the phase and polarization of light (*7*, *16*–*19*). For instance, a circularly polarized (CP) beam passing through a tightly focused or scattering system generates OAM converted by SAM (*6*, *17*). Such SOC localized in the nearfield is derived from the angular momentum (AM) properties of photons (*6*, *16*, *17*), which have a vital role in nearfield optical trapping for nanoparticle-optical manipulation, classification, and deflection (*12*, *20*–*22*), high-resolution optical microscope imaging (*13*, *23*, *24*), optical information storage (*25*, *26*), etc. Current state-of-art methods to detect optical SOIs mostly rely on farfield approaches with compromised fidelity. For example, optical vortices with OAM can be generated by transmitting CP beams through metasurfaces (*19*), or rotations of nanoparticles can be driven according to the AM sign of a sharply focused beam (*27*). While these phenomena qualitatively demonstrate the existence of SOI effects, they fail to provide the quantitative information that needs to be directly extracted in the nearfield. In particular, for a sharply focused structured light beam that enables SOIs near the focal plane, these interactions occur only in the immediate vicinity (nearfield) of the focal plane. As the beam diverges into the farfield, the SAM and OAM quickly become decoupled, thus breaking the SOI states (*17*, *28*). Therefore, accurate evaluation of the SOIs necessitates a comprehensive analysis of both the polarization and spatial characteristics of light in the nearfield. As a result, tackling this challenge becomes an immense task, not only due to the inherent difficulty in precisely characterizing the light amplitude in the nearfield but also owning to the incremented complexity introduced by delving additional DOFs of light in the nearfield.

**Fig. 1.**
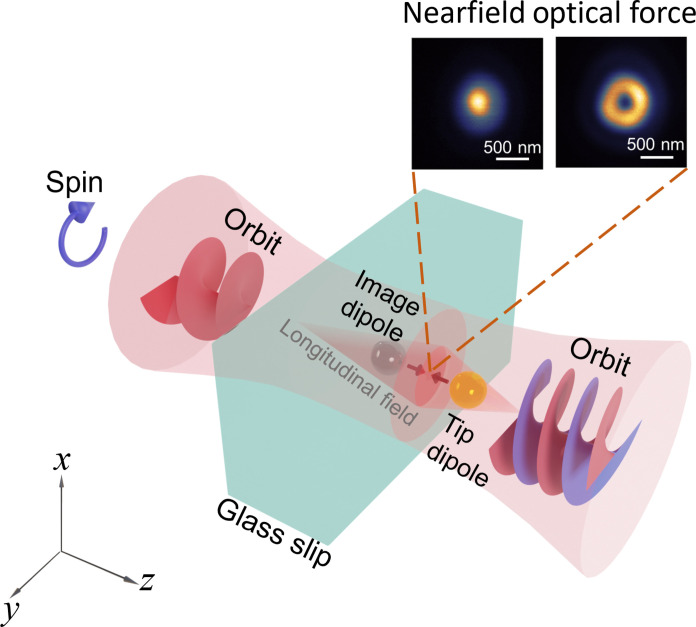
Schematic illustration of a PiFM that measures the subwavelength field intensity distribution of sharply focused structured light. Structured light undergoes spin-orbit conversion (SOC) during sharp focusing, and nearfield optical force distributions are obtained from measurements of the gold-coated tip dipole excitation and its interactions with the glass substrate image dipole using PiFM.

In this work, we propose an approach to achieve accurate evaluation of SOIs directly at the nearfield by using the nearfield optical forces, as illustrated in Fig. 1. We analyze and meticulously characterize the properties of CP vortex beams that are sharply focused using a high–numerical aperture (NA) objective lens in a technique so-called as photoinduced force microscopy (PiFM). PiFM is a nearfield scanning probe force characterization system based on tapping-mode atomic force microscopy (AFM) and an inverted microscope configuration (*29*, *30*). The PiFM-based optical force detection technique provides highly localized detection, enabling imaging of nanomaterials or structures with sub–10 nm spatial resolution (*31*–*34*). It is widely used for structured light characterization (*23*, *35*), optical magnetic force characterization (*31*, *32*, *36*), molecular biochemical sensing (*37*–*39*), and nanoscale infrared analysis in aqueous environments (*40*). PiFM also investigates linear and nonlinear optical properties of nanomaterials (*41*), Raman effect (*42*), chiral effect (*43*, *44*), and two-dimensional (2D) materials (*45*, *46*), with potential applications in biomolecule detection and biotoxin identification (*47*, *48*).

The outstanding resolution and high signal-to-noise ratio (SNR) of the PiFM make it a powerful tool for nanoscale imaging and material characterization. Consequently, PiFM enables the acquisition of high-resolution nearfield optical force maps from sharply focused structured beams. In this study, the measured force maps present unique signatures of the SOIs under various structured light beams, with special emphasis on the longitudinal field components that are only nontrivial in the nearfield. Furthermore, to analyze the optical force map relative to the electric field of the measured beam, we establish a relationship on the basis of a dipole model between optical force and the corresponding electric field. Subsequently, by using the measured nearfield force map and derivations of the field-force relationship, we retrieve the optical field on the focal plane. Last, through comparative analysis of the intensity distribution characteristics across different orders, we realize the distinction of light beams with distinct topological charges. This will serve as an important exploration of complex structured light field analysis at the nanoscale.

## RESULTS

### Principle of sharply focused beams with high-NA focusing

**Fig. 2.**
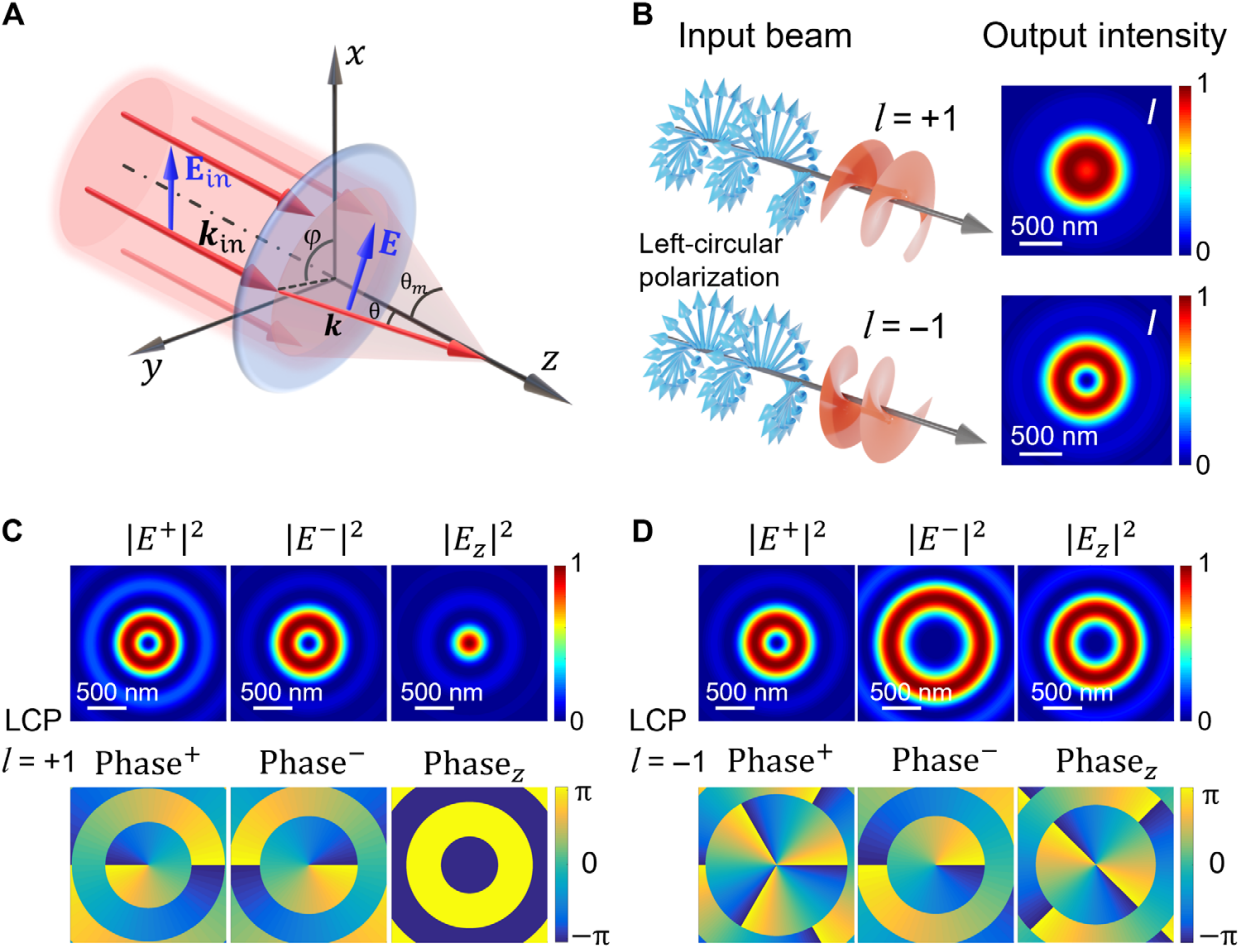
Principle of the tight focusing system and simulation results of left-handed circularly polarized vortex beams. (**A**) Schematic diagram of a high-NA system focusing light. (**B**) The profile of total optical intensity $I={|E_{x}|}^{2}+{|E_{y}|}^{2}+{|E_{z}|}^{2}$ for the sharply focused LCP LG beam with l=±1 topological charge. (**C**) The circular polarization and longitudinal components ${|E^{+}|}^{2},{|E^{-}|}^{2},{|E_{z}|}^{2}$, and phase distributions of the electric field components $\left({\text{Phase}}^{+},{\text{Phase}}^{-},{\text{Phase}}_{z}\right)$ for the LCP LG beam with l=+1, respectively. (**D**) The circular polarization and longitudinal components ${|E^{+}|}^{2},{|E^{-}|}^{2},{|E_{z}|}^{2}$, and phase distributions of the electric field components $\left({\text{Phase}}^{+},{\text{Phase}}^{-},{\text{Phase}}_{z}\right)$ for the LCP LG beam with l=−1, respectively.

In this work, the main task is to investigate the SOIs enabled by sharply focusing CP vortex beams and detect their nearfield property at the focal plane through the PiFM. To achieve this goal, first, we elaborate on the principle of the SOIs induced by sharp focusing (*6*, *7*, *17*, *49*). The beam propagation process of focusing CP vortex beams by a high-NA objective lens, as illustrated in Fig. 2A, constitutes an axially symmetric optical system about the *z* axis. The incident paraxial field ${Ε}_{\text{in}}$ with the wave vector $k_{\text{in}}=ke_{z}$ along the *z* axis is sharply focused by a high-NA objective lens and refracted in the meridional plane (k=2π/λ and $e_{z}$ is the unit vector along the *z* axis). The diffracted angles of the incident beams on the focal plane are expressed by the spherical angles (θ,ϕ) and $\left\{\theta \in \left(0,\theta_{m}\right),\phi \in (0,2\pi )\right\}$, where $\theta_{m}=\text{arcsin}\left(\text{NA}/n_{1}\right)$ is the maximum convergence angle, and $n_{1}$ is the refractive index of the medium in front of the objective lens. Hence, the wave vector $k_{\text{in}}$ is transformed into the nonparaxial wave vector k, denoted by spherical coordinates (θ,ϕ) as

$$k=\left[\begin{array}{c} k_{x}\\ k_{y}\\ k_{z} \end{array}\right]=\left[\begin{array}{c} k_{x}e_{x}\\ k_{y}e_{y}\\ k_{z}e_{z} \end{array}\right]=\left[\begin{array}{c} k\text{sin}\theta \text{cos}\phi e_{x}\\ k\text{sin}\theta \text{sin}\phi e_{y}\\ k\text{cos}\theta e_{z} \end{array}\right]$$ (1)

where $e_{x},e_{y},\text{and}\;e_{z}$ are the unit vectors along the *x*, *y*, and *z* axes, respectively. Therefore, the real-space electric field distribution ${Ε}_{\text{in}}$ of the incident beam after passing through the lens undergoes a Fourier transform into the momentum distribution E in image space. The process can be elucidated by using the Debye-Wolf theory as a geometric rotational transformation $\hat{G}\left(\theta ,\phi \right)$, thereby expressing the focusing field as $E=\sqrt{\text{cos}\theta }\hat{G}\left(\theta ,\phi \right)E_{\text{in}}$ (*6*, *7*, *13*), and

$$\hat{G}\left(\theta ,\phi \right)={\hat{R}}_{z}\left(-\phi \right){\hat{R}}_{y}\left(-\theta \right){\hat{R}}_{z}\left(\phi \right)$$ (2)

where $\sqrt{\text{cos}\theta }$ is the apodization factor (*50*), and ${\hat{R}}_{a}\left(\alpha \right),a=x,y,z$, is the matrix rotated by the angle of α around the a axis.

For the convenience of subsequent description, we use the circular polarization basis relative to the optical axis. On the basis of the transition from the Cartesian basis to the circular basis, the corresponding vector of the complex amplitude electric field under the global basis of the circular polarization (+,−) and longitudinal (z) component is denoted as $E_{c}={\left(E^{+},E^{-},E_{z}\right)}^{T}$. Here $E^{+}\;\text{and}\;E^{-}$ are the right-handed polarization and left-handed polarization electric field components on a circular basis, and $E_{z}$ is the *z* component of the electric field. Hence, the focused field E after this 3D rotation of the global basis is given by (see the Supplementary Materials)

$$\begin{array}{c} E=\sqrt{\text{cos}\theta }\hat{G}\left(\theta ,\phi \right)E_{\text{in}}\\ \;=\sqrt{\text{cos}\theta }\;\left(\begin{array}{ccc} m & -{\mathrm{ne}}^{-2i\phi } & \sqrt{2\mathrm{mn}}e^{-i\phi }\\ -{\mathrm{ne}}^{2i\phi } & m & \sqrt{2\mathrm{mn}}e^{i\phi }\\ -\sqrt{2\mathrm{mn}}e^{i\phi } & -\sqrt{2\mathrm{mn}}e^{-i\phi } & m-n \end{array}\right)\;E_{\text{in}} \end{array}$$ (3)

where $m={\text{cos}}^{2}\left(\theta /2\right)\;\text{and}\;n={\text{sin}}^{2}\left(\theta /2\right)$.

Through Eq. 3, it can be found that the elements other than the main diagonal of the matrix contain the azimuthal vortex factor $e^{\pm i\phi }/e^{\pm 2i\phi }$, which acts for the AM transformation. To illustrate the AM transformation, we analyze the incident light as a CP beam. When the incident light is an RCP beam with helicity σ=+1, the incident field is $E_{\text{in}}={\left(E^{+},0,0\right)}^{T}$, through Eq. 3 that gives the focused field $E_{\text{RCP}}\propto {\left(m,-{\mathrm{ne}}^{2i\phi },-\sqrt{2\mathrm{mn}}e^{i\phi }\right)}^{T}$; in case the incident light is an LCP beam with helicity σ=−1, the incident field is $E_{\text{in}}={\left(0,E^{-},0\right)}^{T}$, and its corresponding electric field at the focal point is denoted as $E_{\text{LCP}}\propto {\left(-{\mathrm{ne}}^{-2i\phi },m,-\sqrt{2\mathrm{mn}}e^{-i\phi }\right)}^{T}$. This shows that the generation of the *z* component of the focused field $E_{z}$ with a helicity σ-dependent azimuthal vortex factor $e^{i\sigma \phi }$ occurs upon the incidence of CP light. At the same time, the CP component of the focused electric field ($E^{+}/E^{-}$), which is in the opposite direction to the polarization of the incident field, produces an azimuthal vortex factor $e^{2i\sigma \phi }$. These components with vortex factors can produce helicity-dependent OAM in the focused field (*7*, *16*, *17*).

In this work, we primarily study the case where the incident fields ${E_{l}^{\sigma }}_{\text{in}}$ are CP vortex beams with topological charge *l* and helicity σ, which can be expressed as ${E_{l}^{\sigma }}_{\text{in}}=e^{\sigma }E_{l}\left(\theta ,\varphi \right),E_{l}\left(\theta ,\varphi \right)=A_{l}\left(\theta \right)e^{\mathrm{il}\varphi }$, where $A_{l}\left(\theta \right)$ is amplitude term. After focusing through the objective lens, the electric field at the focal point can be represented by Eq. 3 as

$$E_{l}^{\sigma }\propto \sqrt{\text{cos}\theta }\hat{G}\left(\theta ,\phi \right)e^{\sigma }E_{l}\left(\theta ,\varphi \right)$$ (4)

Thus, in the case where the incident beam is an RCP vortex beam with ${E_{l}^{+1}}_{\text{in}}$, the focused field is denoted as

$$E_{l}^{+1}={\left(E^{+},E^{-},E_{z}\right)}^{T}\propto {\left[{\mathrm{me}}^{\mathrm{il}\phi },-{\mathrm{ne}}^{i\phi \left(2+l\right)},-\sqrt{2\mathrm{mn}}e^{i\phi \left(1+l\right)}\right]}^{T}$$ (5)

and when the incident beam is an LCP vortex beam with ${E_{l}^{-1}}_{\text{in}}$, the focused field can be written as

$$E_{l}^{-1}={\left(E^{+},E^{-},E_{z}\right)}^{T}\propto {\left[-{\mathrm{ne}}^{i\phi \left(-2+l\right)},{\mathrm{me}}^{\mathrm{il}\phi },-\sqrt{2\mathrm{mn}}e^{i\phi \left(-1+l\right)}\right]}^{T}$$ (6)

This indicates that CP vortex beams will simultaneously have both topological charge l-dependent and helicity σ-dependent azimuthal vortex phase factors when sharply focused, which also accounts for SOIs with spin-to-orbit AM conversion (*17*).

To further illustrate the SOI phenomena in sharply focused CP vortex beams, we next numerically calculate the focused field using the Debye-Wolf theory (*50*, *51*). Here, we consider the incident beams as RCP and LCP vortex beams, and vortex beams in Laguerre Gaussian (LG) mode ${\text{LG}}_{p}^{l}$, where l and p are the angular and radial moduli, respectively, and *l* is also the topological charge of the phase singularity. The LG beams with p=0 in the initial plane at z=0 is denoted as

$$E\left(r,\varphi ,0\right)={\left(\frac{\sqrt{2}r}{w_{0}}\right)}^{|l|}\text{exp}\left(-\frac{r^{2}}{w_{0}^{2}}\right)\text{exp}\left(\mathrm{il}\varphi \right)$$ (7)

where $w_{0}$ is the beam waist. Because a typical lens obeys the sine condition, there exists r=fsinθ (*52*, *53*). Combined with the Debye-Wolf theory, the *x-*, *y-*, and *z*-polarized components of the electric field for the sharply focused RCP and LCP LG beams in cylindrical coordinates at the point $\left(r_{s},\varphi_{s},z_{s}\right)$ of the focusing field can be expressed as

$$\begin{array}{c} E_{\pm ,x}\left(r_{s},\varphi_{s},z_{s}\right)=\frac{-\mathrm{ikf}}{2\sqrt{2}}\int_{0}^{\theta_{\text{max}}}\text{sin}\theta \sqrt{\text{cos}\theta }{\left(\frac{\sqrt{2}f\text{sin}\theta }{w_{0}}\right)}^{|l|}\\ \text{exp}\left[-\frac{f^{2}{\left(\text{sin}\theta \right)}^{2}}{w_{0}^{2}}\right]\cdot e^{\mathrm{ik}\cdot z_{s}\text{cos}\theta }\\ \times \left\{\begin{array}{c} \left(\text{cos}\theta -1\right)\cdot {\left(i\right)}^{l\pm 2}\cdot J_{l\pm 2}\left(k\cdot r_{s}\text{sin}\theta \right)\text{exp}\left[i\left(l\pm 2\right)\varphi_{s}\right]\\ +\left(\text{cos}\theta +1\right)\cdot {\left(i\right)}^{l}\cdot J_{l}\left(k\cdot r_{s}\text{sin}\theta \right)\text{exp}\left(\mathrm{il}\varphi_{s}\right) \end{array}\right\}d\theta \\ E_{\pm ,y}\left(r_{s},\varphi_{s},z_{s}\right)=\frac{-\mathrm{ikf}}{2\sqrt{2}}\int_{0}^{\theta_{\text{max}}}\text{sin}\theta \sqrt{\text{cos}\theta }{\left(\frac{\sqrt{2}f\text{sin}\theta }{w_{0}}\right)}^{|l|}\\ \text{exp}\left[-\frac{f^{2}{\left(\text{sin}\theta \right)}^{2}}{w_{0}^{2}}\right]\cdot e^{\mathrm{ik}\cdot z_{s}\text{cos}\theta }\\ \times \left\{\begin{array}{c} \frac{\left(\text{cos}\theta \pm 1\right)}{2}\cdot {\left(i\right)}^{l\pm 2}\cdot J_{l\pm 2}\left(k\cdot r_{s}\text{sin}\theta \right)\cdot \text{exp}\left[i\left(l\pm 2\right)\varphi_{s}\right]\\ +\left[i\left(\frac{\text{cos}\theta \pm 1}{2}\right)\right]\cdot {\left(i\right)}^{l}\cdot J_{l}\left(k\cdot r_{s}\text{sin}\theta \right)\text{exp}\left(\mathrm{il}\varphi_{s}\right) \end{array}\right\}\;d\theta \\ E_{\pm ,z}\left(r_{s},\varphi_{s},z_{s}\right)=\frac{-\mathrm{ikf}}{\sqrt{2}}\text{exp}\left(l\pm 1\right)i\varphi_{s}\int_{0}^{\theta_{\text{max}}}{\left(\frac{\sqrt{2}f\text{sin}\theta }{w_{0}}\right)}^{|l|}\\ \text{exp}\left[-\frac{f^{2}{\left(\text{sin}\theta \right)}^{2}}{w_{0}^{2}}\right]{\text{sin}}^{2}\theta \sqrt{\text{cos}\theta }\cdot e^{\mathrm{ik}\cdot z_{s}\text{cos}\theta }\\ \times {\left(i\right)}^{l\pm 1}\cdot J_{l\pm 1}\left(k\cdot r_{s}\text{sin}\theta \right)d\theta \end{array}$$ (8)

where $J_{N}\left(x\right)$ is an *N*-order Bessel function of the first kind. The subscripts “+” and “−” indicate that the polarization of the incident beams is RCP and LCP, respectively.

Meanwhile, we can calculate the total intensity of the sharply focused RCP and LCP LG beams as follows

$$I={|E_{\pm ,x}\left(r_{s},\varphi_{s},z_{s}\right)|}^{2}+{|E_{\pm ,y}\left(r_{s},\varphi_{s},z_{s}\right)|}^{2}+{|E_{\pm ,z}\left(r_{s},\varphi_{s},z_{s}\right)|}^{2}$$ (9)

Next, we study the properties of sharply focused RCP/LCP LG beams with σ=±1 and l=±1, in which cases the total AM is either ∣L∣=2 or ∣L∣=0, based on the above formulas. In the numerical calculations, we set λ=633 nm, $w_{0}=3$ mm, NA=1.3, $n_{1}=1.515$, and f=1.8 mm. Figure 2B shows the distribution of total intensity of the LCP LG beams with σ=−1 and l=±1 in the focal plane, respectively, we can find that the total intensity profile is bright center shaped when l=+1 (total AM ∣L∣=0), while there is donut shaped when l=−1 (total AM ∣L∣=2). Meanwhile, the intensity of circular polarization (+,−) and longitudinal z components and the corresponding phase distributions in the focal plane are depicted in Fig. 2 (C and D). It indicates that the distribution of dark and bright spots at the center of the optical intensity in Fig. 2B can be solely attributed to the longitudinal z component of the focused fields. Furthermore, in the phase distribution of the circular polarized and longitudinal components $\left({\text{Phase}}^{+},{\text{Phase}}^{-},{\text{Phase}}_{z}\right)$ of the LCP LG beam carrying l=+1, as illustrated in Fig. 2C, the corresponding phase changes are −2π, 2π, and 0, respectively, aligning with the vortex factor contained in each component of Eq. 6. Likewise, as depicted in Fig. 2D, the phase changes for each component of the LCP LG beam with l=−1 are −6π, −2π, and −4π, respectively. These changes precisely match the vortex factors associated with each component in Eq. 6. By analogy, we anticipate analogous behavior for the RCP LG beams with σ=+1 and l=±1, in which the intensity and phase distribution are appended in fig. S1. When l=+1, the total intensity distribution manifests as a donut shape, and the phase distributions of phase changes are 2π, 6π, and 4π, respectively. Conversely, when l=−1, the total intensity distribution presents as a solid-center shape, and the phase distributions of phase changes are −2π, 2π, and 0, respectively. These distributions match the vortex factor contained in each component of Eq. 5. The directions of rotation about the phase profiles depend on the helicity-dependent topological charge superimposed on the topological charge carried by the beam itself. Therefore, the interaction of the vortex factor carried by the LG beam itself with the helicity-dependent vortex factor produced by the focusing process results in a different longitudinal component of the focusing field, which determines the difference between the dark (with singularity) and bright (without singularity) spots at the vicinity of the beam axis.

Through the wave spectrum analysis and numerical calculations above, we can find that, due to the helicity-dependent topological charge generated during the focusing process, the longitudinal component after sharp focusing undergoes AM conversion from SAM to OAM. As a result, it grants a unique signature of the structured light intensity profile: When the total AM of the incident beam is ∣L∣=2, the total OAM of the focused beam is nonzero, which induces a singularity at the beam axis and makes a donut-shape beam spot; when the total AM of the incident beam is ∣L∣=0, the total OAM of the focused beam is zero, which compensates the singularity at the beam axis and makes a bright center beam spot.

### Principle of optical force detection

**Fig. 3.**
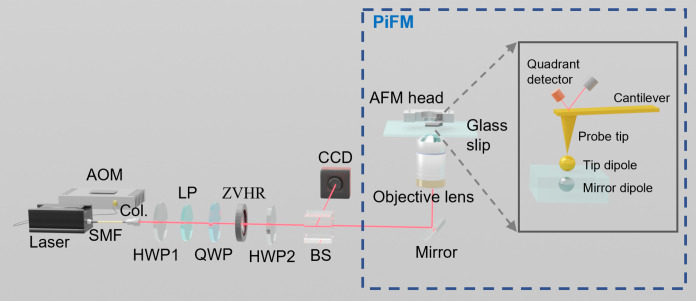
Experimental setup for the detection of sharply focused vector beams and vortex beams by PiFM. AOM, acoustic-optic modulator; SMF, single-mode fiber; Col., collimator; HWP, half-wave plate; LP, linear polarizer; QWP, quarter-wave plate; ZHVR, zero-order half-wave vortex retarder; BS, beam splitter; CCD, charge-coupled device.

Because the sharp focusing enabled by a high-NA objective lens induces complex subwavelength optical field structures at the focal plane, in principle, they can only be accurately detected by nearfield characterization methods. In this work, we use the PiFM system for high-fidelity nearfield detection through the nearfield optical force. As shown in Fig. 3, we produce the desired structured light through a series of external optics and then direct the light to the PiFM instrument. Inside the PiFM, the incident beam is focused through a glass slide to a nanoprobe attached to an AFM head, which can detect the force between the probe tip and the glass slide. The total force exerted on the probe, naturally, may include both the photoinduced and non-photoinduced forces. Here, the non-photoinduced forces include all the chemical, Casimir, meniscus, atomic, and van der Waals forces, while the photoinduced force originates from the Lorentz force induced by the probe tip excited by the incident electromagnetic field (*30*, *35*, *36*, *54*). Electromagnetic multipole theory offers a more concise and intuitive approach to analyzing the optical properties of nanostructures (*55*). This theory significantly simplifies the analysis of complex electromagnetic field problems by representing arbitrary distributions as a superposition of multipole contributions (*56*, *57*). Specifically, complex nearfield forces can be understood through multipole analysis, which includes electromagnetic dipole, quadrupole, and higher-order multipole modes. Typically, the optical response of simple nanoparticles under light illumination can be described using dipole approximation theory. For larger particles or more complex structured light fields, higher-order multipole modes become significant (*58*–*60*). For example, tunable transverse optical forces can be generated using multipolar interactions of single elongated particles (*60*). In our study, we consider the probe tip as a subwavelength nanoparticle that may exhibit both electric and magnetic dipole responses, while contributions from higher-order multipoles can be reasonably neglected (*32*, *35*). The corresponding electric and magnetic dipole moments are denoted as $p_{\text{tip}}$ and $m_{\text{tip}}$, respectively. Under the dipole approximation, the time-averaged photoinduced force applied to the probe tip is (*35*, *36*)

$$\begin{array}{c} \langle F_{\text{pho}}\rangle =\frac{1}{2}\\ \text{Re}\left\{{\left[\nabla E^{\text{loc}}\left(r\right)\right)}^{*}\cdot p_{\text{tip}}+\mu_{0}{\left[\nabla H^{\text{loc}}\left(r\right)\right]}^{*}\cdot m_{\text{tip}}-\mu_{0}\frac{{\mathrm{ck}}^{4}}{6\pi }\left[p_{\text{tip}}\times {\left(m_{\text{tip}}\right)}^{*}\right]\right\} \end{array}$$ (10)

Here, $E^{\text{loc}}$ and $H^{\text{loc}}$ are the local electric and magnetic fields, respectively; $\mu_{0}$ is the free-space permeability, *c* is the speed of light in vacuum, k is the free-space wave number, and the superscript * denotes complex conjugation. Here, the first term on the right side of Eq. 10 indicates the time-averaged force $\langle F_{e}\rangle$ caused by the electric dipole, the second term indicates the time-averaged force $\langle F_{m}\rangle$ caused by the magnetic dipole, and the last term indicates the time-averaged force $\langle F_{e-m}\rangle$ caused by the interacted electric dipole and magnetic dipole.

In our experiments, we use a gold-coated probe, where the electric dipole response dominates at the optical frequency, and the magnetic dipole induced on the tip can be ignored, implying that the force resulting from the magnetic dipole can be neglected. Consequently, Eq. 10 can be simplified as follows (*36*)

$$\langle F_{\text{pho}}\rangle \approx \langle F_{e}\rangle =\frac{1}{2}\text{Re}\left\{{\left[\nabla E^{\text{loc}}\left(r\right)\right]}^{*}\cdot p_{\text{tip}}\right\}$$ (11)

In addition, in the current PiFM system settings, we use the tapping mode to control the movement of the cantilever over the sample surface with the probe tapping along z direction; thus, only the longitudinal component of the time-averaged photoinduced force is detected in our specific PiFM system. Therefore, the longitudinal optical force is expressed as

$$\langle F_{\text{pho},z}\rangle =\frac{1}{2}\text{Re}\left\{\sum_{j}{\left[\partial_{z}E_{j}^{\text{loc}}\left(r\right)\right]}^{*}\cdot p_{j}\right\},j=x,y,z$$ (12)

that is

$$\begin{array}{c} \langle F_{\text{pho},z}\rangle =\frac{1}{2}\\ \text{Re}\left\{{\left[\frac{\partial E_{x}^{\text{loc}}\left(r\right)}{\partial z}\right]}^{*}\cdot p_{\text{tip},x}+{\left[\frac{\partial E_{y}^{\text{loc}}\left(r\right)}{\partial z}\right]}^{*}\cdot p_{\text{tip},y}+{\left[\frac{\partial E_{z}^{\text{loc}}\left(r\right)}{\partial z}\right]}^{*}\cdot p_{\text{tip},z}\right\} \end{array}$$ (13)

Here, $\partial_{z}$ is the partial derivative to the *z* spatial coordinate; j=x,y,z are the Cartesian coordinates. Where the local electric field $E^{\text{loc}}\left(r\right)$ at the probe tip is derived from the incident field and the scattered field of the image dipole, that is

$$E^{\text{loc}}\left(r_{\text{tip}}\right)=E^{\text{in}}\left(r_{\text{tip}}\right)+E_{\text{img}\to \text{tip}}^{\text{sca}}\left(r_{\text{tip}}\right)$$ (14)

Here, $E^{\text{in}}\left(r_{\text{tip}}\right)$ is the incident field at the tip of the probe; $E_{\text{img}\to \text{tip}}^{\text{sca}}\left(r_{\text{tip}}\right)$ is the scattered field by the image dipole.

It is to be noted that, within an acceptable approximation, considering only the polarization terms below the second order (*35*, *36*), the longitudinal component of the time-averaged force is obtained as

$$\begin{array}{c} \langle F_{\text{pho},z}\rangle =-\frac{3}{8{\pi \varepsilon }_{0}d^{4}}\\ \text{Re}\left\{\begin{array}{c} \left(\alpha_{\text{tip},\mathrm{xx}}E_{x}^{\text{focal}}\right)\cdot {\left(\alpha_{\text{img},\mathrm{xx}}E_{x}^{\text{focal}}\right)}^{*}+\left(\alpha_{\text{tip},\mathrm{yy}}E_{y}^{\text{focal}}\right)\cdot {\left(\alpha_{\text{img},\mathrm{yy}}E_{y}^{\text{focal}}\right)}^{*}\\ -2\left(\alpha_{\text{tip},\mathrm{zz}}E_{z}^{\text{focal}}\right)\cdot {\left(\alpha_{\text{img},\mathrm{zz}}E_{z}^{\text{focal}}\right)}^{*} \end{array}\right\} \end{array}$$ (15)

where d is the distance between the image and tip dipoles; $\alpha_{\text{tip},\mathrm{ij}}$ and $\alpha_{\text{img},\mathrm{ij}}$ (i,j=x,y,z) are the electric polarizabilities of the tip and the image along the x,y_,_ and z axes, respectively; and $E_{x}^{\text{focal}},E_{y}^{\text{focal}},\text{and}\;E_{z}^{\text{focal}}$ are the electric field components of the focused field at the probe detection location, respectively.

We assume azimuthal symmetry in the *x*-*y* transverse plane, i.e., $\alpha_{r}=\alpha_{\mathrm{xx}}=\alpha_{\mathrm{yy}}$; thus, Eq. 15 can be further expressed as

$$\begin{array}{c} \langle F_{\text{pho},z}\rangle \approx -\frac{3}{8{\pi \varepsilon }_{0}d^{4}}\\ \text{Re}\left(\alpha_{\text{tip},r}\alpha_{\text{img},r}{\left|E_{r}^{\text{focal}}\right|}^{2}-2\alpha_{\text{tip},z}\alpha_{\text{img},z}{\left|E_{z}^{\text{focal}}\right|}^{2}\right) \end{array}$$ (16)

where ${\left|E_{r}^{\text{focal}}\right|}^{2}={\left|E_{x}^{\text{focal}}\right|}^{2}+{\left|E_{y}^{\text{focal}}\right|}^{2}$. This equation indicates that the measured longitudinal optical force results from contributions of both the transverse and longitudinal electric fields. By combining Eq. 16 with Eq. 8, the force-field relation can be established to analyze the SOIs based on the force measurement.

### The PiFM characterization

PiFM instrument is a kind of microscope that combines an inverted objective system with an AFM head. PiFM characterizes the optical force distribution of the focused beam, mainly through the probe tip-cantilever system in the AFM head to detect and record the force generated by the motion of the probe under the beam illumination and extract the photoinduced force through a lock-in amplification system. Specifically, the force signals detected by the probe include both photoinduced and non-photoinduced forces. To distinguish the photoinduced force, the incident laser beam needs to be amplitude-modulated at a frequency of $f_{m}$, and then a series of optics are used to set up the target beam, after which the beam is focused on the probe tip and the sample through a high-NA objective lens. At this moment, the cantilever generates motions with respect to $f_{m}$ and the motion signals are analyzed and detected by a quadrant detector, after which a lock-in amplifier demodulates the signals from the quadrant detector at a specific frequency to obtain the photoinduced force signal. In our PiFM setting, the photoinduced force signal is obtained from the cantilever at the second mechanical resonance frequency $f_{2}$, and the AFM morphology is obtained from the cantilever at the first mechanical resonance frequency $f_{1}$, which is laser-modulated using the sum or difference frequency of the sideband detection modes ($f_{m}=f_{1}+f_{2}$ or $f_{m}=f_{1}-f_{2}$). Thus, the lock-in amplifier tunes the demodulation frequency near $f_{2}$ to obtain the photoinduced force profile.

The schematic diagram of the experimental setup adopted to characterize the sharply focused RCP and LCP LG beam is shown in Fig. 3. The laser beam at 633 nm is modulated in amplitude using an acoustic-optic modulator at the frequency $f_{m}$ and then passed through an objective coupling system into a single-mode fiber, which is connected to a fiber collimator to obtain a collimated fundamental mode Gaussian beam. The first half-wave plate and a linear polarizer are used to modulate the intensity of the incident beam and facilitate the arbitrary generation of linear polarization. Following this setup, a quarter-wave plate is then added when needed to convert the incident linearly polarized beam into an RCP or LCP beam, and the handedness of the CP beam can be easily adjusted via its fast axis direction. A zero-order half-wave vortex retarder (ZHVR) is used to generate vector beams and vortex beams. Specifically, a radially polarized beam is generated when the incident beam is linearly polarized and parallel to the fast axis of the ZHVR, and an azimuthally polarized beam (APB) is generated when the incident beam is linearly polarized and vertical to the fast axis of the ZHVR. In addition, a CP beam passing through the ZHVR produces an LG mode vortex beam carrying +l or −l topological charge. The sign (+/−) is contingent upon the input polarization of the beam, and the value of l associated with the order of ZHVR. Here, the order of the used ZVHR (from Thorlabs, model WPV10-633) is l=+1. When an RCP beam traverses the ZHVR, an LG vortex beam with a topological charge of +1 is generated and the polarization state becomes LCP; conversely, when an LCP beam passes through the ZHVR, an LG vortex beam with a topological charge of −1 is generated and the polarization state transforms to RCP. Following this setup, a second half-wave plate is introduced as required to alter the polarization state of the vortex beam generated by the ZHVR, transitioning it from RCP/LCP to LCP/RCP. A beam splitter propagates the generated vector beams or CP vortex beams into the charge-coupled device (CCD) camera and PiFM systems to obtain the farfield intensity distribution and nearfield optical force distribution, respectively. The beam entering the PiFM system is focused onto the AFM head through a high-NA oil immersion objective (NA = 1.3, the refractive index of oil is 1.515). Then, the gold-coated probe (ACTGG from AppNano) with $f_{1}$ and $f_{2}$ at 1471 and 242 kHz is mounted on the AFM head, and it is subjected to AC tapping mode along the longitudinal direction. Meanwhile, the modulation frequency of the laser has $f_{m}=f_{1}+f_{2}=1713\;\text{kHz}$. The cantilever is driven at its first mechanical resonance mode, and the PiFM signal is detected at the second mechanical resonance; thus, the nearfield photoinduced force distribution of the optical field is obtained.

**Fig. 4.**
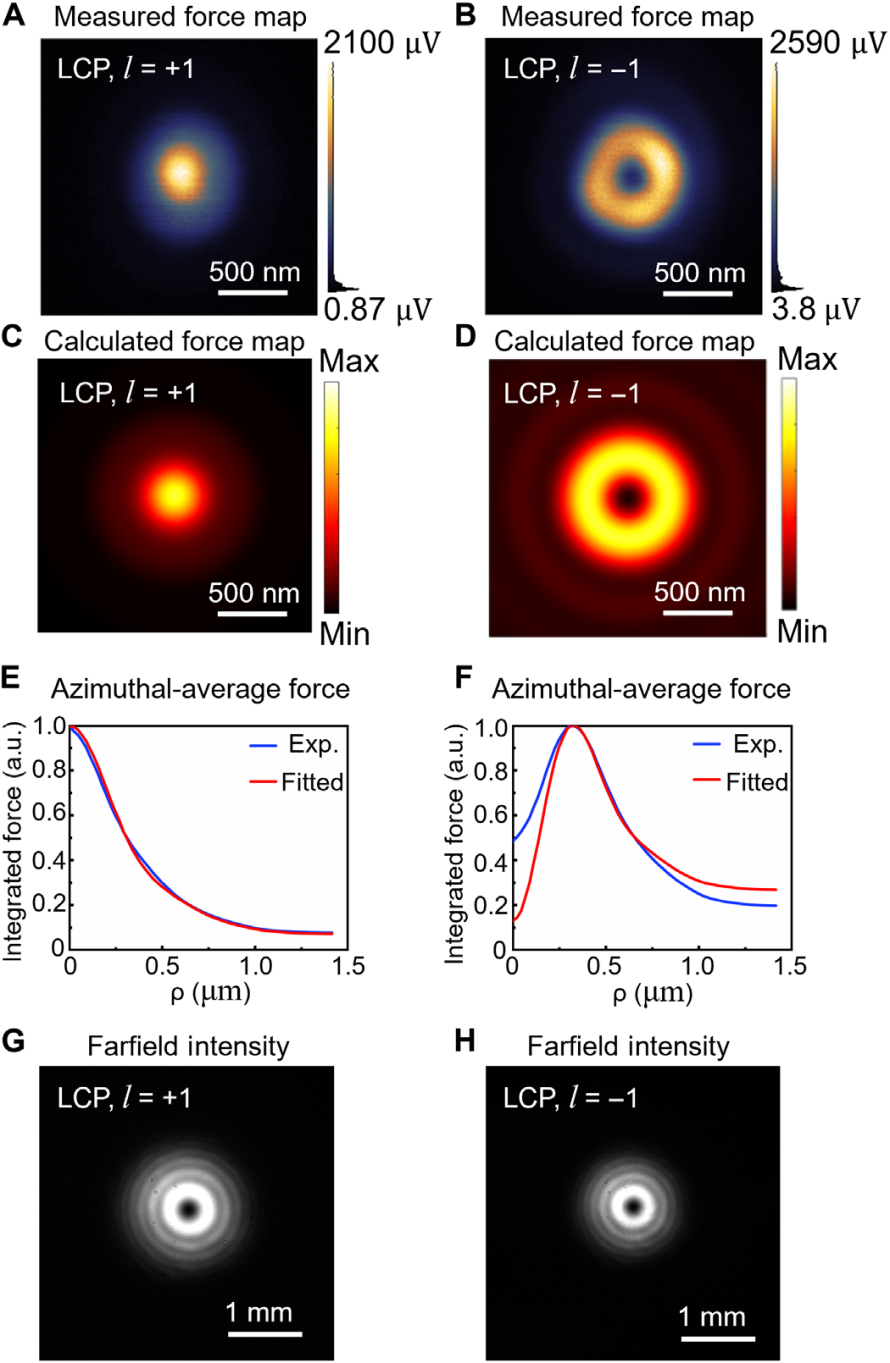
Nearfield force and farfield intensity distributions and fitted results of left-handed circularly polarized vortex beams. PiFM direct nearfield force measurement distributions of (**A**) LCP LG beams with l=+1 topological charge and (**B**) LCP LG beams with l=−1 topological charge; optical force distributions obtained by numerical model fitting for (**C**) the LCP LG beam with l=+1 topological charge and (**D**) the LCP LG beam with l=−1 topological charge; experimental results and fitted numerical model of azimuthal-average force versus radial distance from the beam axis ρ for (**E**) LCP LG beam with l=+1 topological charge and (**F**) LCP LG beam with l=−1 topological charge. Distributions of intensity detected by farfield CCD for (**G**) LCP LG beam with l=+1 topological charge and (**H**) LCP LG beam with l=−1 topological charge. a.u., arbitrary units.

Therefore, using the experimental optical system outside the PiFM optical path depicted in Fig. 3, we generate LCP LG beam with l=+1, LCP LG beam with l=−1, RCP LG beam with l=+1, and LCP LG beam with l=−1, respectively. After that, the generated specific beams are transmitted to the PiFM system. Following objective lens focusing and probe scanning detection, we obtain the force maps of the sharply focused RCP and LCP LG beams, and the full results of the force measurements are shown in fig. S3. To further analyze the relationship between experimental optical force measurements and the focused light field, we fit numerical models to experimental data. This analysis aims to assess the contributions of transverse and longitudinal fields to the measured longitudinal optical force (details are provided in the Supplementary Materials). Here, in Fig. 4, we present the final comparison between the experiment and fitted model for a typical case with LCP LG beams with l=±1. The force maps measured in the nearfield in Fig. 4 (A and B) show a bright center shape and a donut-shaped profile with subwavelength features. These measured force maps indicate nanoscale resolution. Compared with the CCD images obtained before focusing on the PiFM system (as depicted in Fig. 4, G and H), the LCP LG beams with equal polarization helicity and different topological charges exhibit a symmetric donut distribution in the farfield. Figure 4 (C and D) illustrates the optical force distribution obtained using the numerical model. To eliminate the effect of anisotropy in the experimentally measured force maps, we average the forces over the azimuthal angle φ to obtain the experimental force data and the fitted data versus the radial distance to the beam axis ($\rho =\sqrt{x^{2}+y^{2}}$), as shown in Fig. 4 (E and F). Moreover, the corresponding transverse light field distribution and longitudinal light field distribution retrieved are shown in fig. S5 (A and B), respectively. In addition, please refer to the Supplementary Materials for the experimental optical force distributions, farfield distributions, and optical force distributions obtained from the fitted model and the retrieved transverse and longitudinal light field distribution for the RCP LG beams.

## DISCUSSION

Here, we provide in-depth analysis and evaluation to interpret the experiment and modeling results. First, the proposed method based on the optical force measurement can acquire the longitudinal nearfield component with superior accuracy, which is essential to investigate the nearfield SOC with high fidelity. It is imperative to underscore that the longitudinal field component stands out as a unique characteristic of sharply focused structured light. As shown in Fig. 4 (A and B), this component induces distinct topology features in the force maps at the nearfield, such as a bright center spot or a donut-shaped spot, respectively, that are not observable at the farfield. In particular, here the generation of the longitudinal nearfield that carries OAM is, actually, the reason for the SOC that partially converts the SAM into OAM. We emphasize that this SOC process not only maximizes the utilization of all three spatial dimensions for AM conversion but also accomplishes it in an ultracompact manner at the subwavelength nanoscale. Therefore, the proposed method can be promising for future optical computing devices founded on nearfield SOC. Such devices could potentially harness an expanded information basis, leading to a substantial enhancement in computational power (*61*).

Second, thanks to the superior SNR of the optical force measurement, a profound understanding of the actual OAM mode at the nearfield becomes feasible through the accurate profile of the measured donut shape force map. Crucially, determining the topological charges of the OAM beams stands out as a central task for all OAM applications. However, most of the mainstream OAM detection methods used to date rely heavily on farfield interference, rendering them impractical for nearfield applications.

**Fig. 5.**
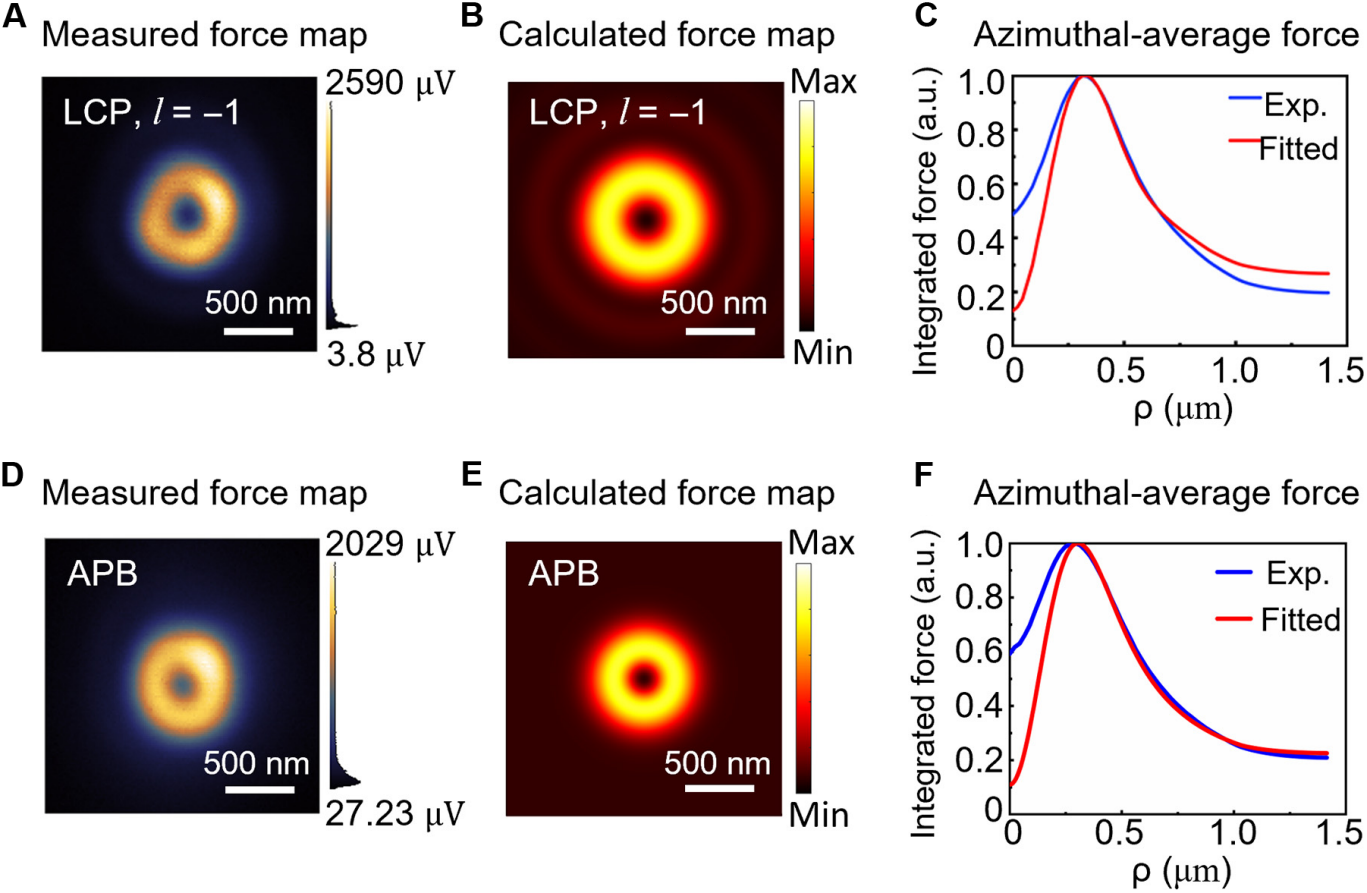
Nearfield force profiles and fitted results for beams with different topological charge numbers. (**A**) PiFM direct nearfield force measurement of LCP LG beam with l=−1 topological charge. (**B**) Optical force distribution of the LCP LG beam with l=−1 topological charge obtained by numerical model fitting. (**C**) Experimental results and fitted numerical model of azimuthal-average force versus radial distance from the beam axis ρ for LCP LG beam with l=−1 topological charge. (**D**) PiFM direct nearfield force measurement of APB. (**E**) Optical force distribution of the APB obtained by numerical model fitting. (**F**) Experimental results and fitted numerical model of azimuthal-average force versus radial distance from the beam axis ρ for APB.

In principle, OAM beams with different topological charges can manifest different donut shapes, where the ratio between the radius of the dark center and outer ring can be varied. The higher order of the OAM beam induces a relatively larger dark center in the donut-shaped beam profile (*62*). As shown in Fig. 5, we measure the nearfield optical force distributions of the APB using the PiFM nearfield system (as shown in Fig. 5, D and E), and we fit two different measured sharply focused structured light beams (LCP LG beams with l=−1 and APB beams) with numerical models and then accurately characterize the effective OAM. Expectedly, the focused LCP LG beam with the dominant l=2 OAM mode profile exhibits a bigger center than the focused APB with l=1 OAM mode profile.

Building upon the modeling of the meticulously measured high-resolution, high-SNR force maps, our experiment enables a quantitative determination of the precise SOC. Once the numerical models are fitted to the measured force maps, a comprehensive understanding of the electric and magnetic nearfields can be obtained.

Moreover, in this work we endeavor to analyze the SOI effect from sharp focusing by using the PiFM, which serves as an efficient tool to characterize the transition from the SAM in the transverse farfield to the OAM in the longitudinal nearfield. In this approach, the incident beam already has a well-defined farfield. In the cases where the farfield features cannot be conveniently acquired, the tangible methods to distinguish the symmetric cases (e.g., the RCP LG beam with l=1 and the LCP LG beam with l=−1) may be using nearfield phase measurement by scattering scanning near-field optical microscopy (*63*, *64*) or using chiral probes (*65*, *66*). This would be an intriguing direction to be researched in the future.

Last, we emphasize that the current experiment only indicates one representative example of SOC characterization by the PiFM system. The SOC can be enabled by many means, such as metasurfaces, nanoparticles, or special beams (*7*, *28*, *67*). While we use the PiFM to investigate the nearfield SOC as a relatively simple example of sharp focusing, there is no constraint on extending this method to other nearfield SOC scenarios. The superior performance of the PiFM, marked by its high SNR and resolution, offers the prospect of analyzing intricate nearfield properties through optical force, complemented by rigorous theoretical analysis to interpret the force-field relationship. Drawing from the insights gained in this initial example, we anticipate that nearfield optical force can emerge as a crucial tool for investigating a broader array of more complex light field manipulations in the near future.

In summary, we use the PiFM as a powerful tool to analyze the SOI properties of sharply focused CP OAM beams with remarkable nanoscale precision. Leveraging experimental nearfield optical force images and farfield CCD images, we discern distinct features in the measured force maps in the nearfield, which are attributed to the conversions between the SAM (σ) and OAM (l) of the beams. Specifically, when the total AM ∣L∣=2, the force maps exhibit a donut-shaped feature, while, for ∣L∣=0, the force maps display a bright spot shape. The high-resolution advantage of nearfield detection allows us to directly observe these features at subwavelength scales and understand the non-paraxial characteristics of light. Furthermore, by fitting the measured force map to the numerical modal of the SOI beams, we are able to quantitatively retrieve the optical field distribution corresponding to the experimental optical force. This method defines the essential step toward interrogating the intricate photon DOFs with coupled dimensions at the nearfield, laying an important foundation for future advancements in nanophotonic research with high-speed, dense, and all-optical devices and manipulations.

## MATERIALS AND METHODS

For the measurements of the nearfield characteristics of CP vortex beams, we combined a free-space optical setup with a PiFM system, as illustrated in Fig. 3. The main component is the ZHVR (from Thorlabs, model WPV10-633, with l=1). The sharp focusing of the CP vortex beams is achieved by an Olympus high-NA oil-immersion objective. There, the power of the final incident beam into the PiFM system is about 200 μW.

The PiFM system for the experiment is a commercial PiFM VisaScope from Molecular Vista Inc., which includes a commercial AFM detection head. Through using the integrated lock-in mechanism and the AFM detection, the photoinduced force signal is extracted. The gold-coated probe model ACTGG (from AppNano) is used in AC tapping mode, and the detection frequency used in the experiment was 242 kHz.

## Supplementary Materials

### This PDF file includes:

Supplementary Text Figs. S1 to S6 References
