# Supplementary material for: Nearfield observation of spin-orbit interactions at nanoscale using photoinduced force microscopy
Source: Sci Adv. 2024 Dec 20;10(51):eadp8460. doi: 10.1126/sciadv.adp8460 (PMC12697560; doi:10.1126/sciadv.adp8460)
Supplement: Supplementary file 1 — Supplementary Text Figs. S1 to S6 References [file sciadv.adp8460_sm.pdf]

Science Advances
AAAS

Supplementary Materials for

Nearfield observation of spin-orbit interactions at nanoscale using photoinduced force microscopy

Yajuan Dong *et al.*

Corresponding author: Jinwei Zeng, zengjinwei@hust.edu.cn; Jian Wang, jwang@hust.edu.cn

Sci. Adv. **10**, eadp8460 (2024)
DOI: 10.1126/sciadv.adp8460

This PDF file includes:

Supplementary Text
Figs. S1 to S6
References

Supplementary Text

Theory of high-resolution objective lens focusing systems using the Debye-Wolf method

We can use the Debye-Wolf theory to express the process as a geometric rotational transformation $\widehat{G}(\theta,\phi)$ (*6*, *7*, *13*).

The system is an axisymmetric optical system concerning the z-axis. For the convenience of subsequent description, we use the circular polarization basis relative to the optical axis. In the Cartesian coordinates, the basis vectors are represented by $(\mathbf{e}_x,\mathbf{e}_y,\mathbf{e}_z)$, and the corresponding vector of complex amplitude electric field is represented by $\mathbf{E}_C=(E_x,E_y,E_z)^T$. The basis vectors and electric field components in the circular polarization basis are expressed as

$$\begin{aligned}\mathbf{e}^{\pm}&=\frac{\mathbf{e}_x\pm i\mathbf{e}_y}{\sqrt{2}}\\E^{\pm}&=\frac{E_x\mp iE_y}{\sqrt{2}}\end{aligned},\tag{S1}$$

where E^+,E^- are the right-handed polarization and left-handed polarization electric field components in the circular basis, respectively. Therefore, the corresponding vector of the complex amplitude electric field under the global basis of the circular polarization $(+,-)$ and longitudinal (z) component is denoted as $\mathbf{E}_c=(E^+,E^-,E_z)^T$. Based on this, the transition from the Cartesian basis to the circular basis will undergo a unitary transformation, denoted as

$$\mathbf{E}_C=\widehat{U}\mathbf{E}_c,\widehat{U}=\frac{1}{\sqrt{2}}\begin{pmatrix}1&1&0\\i&-i&0\\0&0&\sqrt{2}\end{pmatrix}.\tag{S2}$$

Based on this convention, the geometric rotational transformation $\widehat{G}(\theta,\phi)$ experienced by the focusing process is expressed as

$$\widehat{G}(\theta,\phi)=\widehat{U}^{\dagger}\widehat{R}_z(-\phi)\widehat{R}_y(-\theta)\widehat{R}_z(-\phi)\widehat{U},\tag{S3}$$

where $\widehat{R}_a(\alpha),a=x,y,z$ is the matrix rotated by the angle of α around the a-axis. Hence, the focused field $\mathbf{E}$ undergoing this three-dimensional rotation is indicated as

$$\begin{aligned}\mathbf{E}&=\sqrt{cos\theta}\,\widehat{G}(\theta,\phi)\mathbf{E}_{\text{in}}\\&=\sqrt{cos\theta}\begin{pmatrix}m&-ne^{-2i\phi}&\sqrt{2mn}e^{-i\phi}\\-ne^{2i\phi}&m&\sqrt{2mn}e^{i\phi}\\-\sqrt{2mn}e^{i\phi}&-\sqrt{2mn}e^{-i\phi}&m-n\end{pmatrix}\mathbf{E}_{\text{in}}\end{aligned},\tag{S4}$$

where $\sqrt{cos\theta}$ is the apodization factor (*50*), and $m=cos^2(\theta/2),n=sin^2(\theta/2)$.

Numerical derivation of focused circularly polarized Laguerre Gaussian beams based on Debye-Wolf theory

According to Debye-Wolf theory (*50*), the electric field near the focal spot in cylindrical coordinates can be expressed as

$$\mathbf{E}=\frac{-ikf}{2\pi}\int_0^{\theta_{\max}}\int_0^{2\pi}\mathbf{A}(\theta,\varphi)\mathrm{e}^{i\mathbf{k}\cdot\mathbf{r}_s}\sin\theta d\varphi d\theta\,, \tag{S5}$$

where $k=2\pi n_1/\lambda$ is the wave number, λ is the wavelength of the incident beam, and n_1 is the refractive index of the medium in front of the objective lens, f is the focal length, θ varies from 0 to $\theta_{\max}$, $\mathbf{k}$ is the nonparaxial wavevector, $\mathbf{r}_s$ designates the position of the observation point in the image space. And $\mathbf{A}(\theta,\varphi)$ is the field strength factor, denoted as

$$\mathbf{A}(\theta,\varphi)=T(\theta)E(\theta,\varphi)\mathbf{P}(\theta,\varphi)\,, \tag{S6}$$

in which, $T(\theta)=\sqrt{\cos\theta}$ is the apodization function of the lens, $E(\theta,\varphi)$ is the incident field at the entrance pupil, and $\mathbf{P}(\theta,\varphi)$ is the electric polarization vector in the focused field.
Here, we consider the incident beams as circular polarization (CP) vortex beams, and vortex beams in Laguerre Gaussian (LG) mode LG_p^l, where l, p are the angular and radial moduli, respectively, and *l* is also the topological charge of the phase singularity. The LG beams in the initial plane at $z=0$ is denoted as

$$\mathrm{E}(r,\varphi,0)=(\frac{\sqrt{2}r}{w_0})^{|l|}\mathrm{LG}_p^l(\frac{2r^2}{w_0^2})\exp(-\frac{r^2}{w_0^2})\exp(il\varphi)\,, \tag{S7}$$

where w_0 is the beam waist, considering $p=0$, the Eq. S7 can be expressed as

$$\mathrm{E}(r,\varphi,0)=(\frac{\sqrt{2}r}{w_0})^{|l|}\exp(-\frac{r^2}{w_0^2})\exp(il\varphi)\,. \tag{S8}$$

Since a typical lens obeys the sine condition, there exists $r=f\sin\theta$ (*52*, *53*), the expression for the electric field of a sharply focused circular polarization Laguerre Gaussian (CP LG) beam in cylindrical coordinates can be expressed as:

$$E(r_s,\varphi_s,z_s)=\frac{-ikf}{2\pi}\int_0^{\theta_{\max}}\int_0^{2\pi}\sin\theta\sqrt{\cos\theta}(\frac{\sqrt{2}\mathrm{f}\sin\theta}{w_0})^{|l|}\exp(-\frac{\mathrm{f}^2(\sin\theta)^2}{w_0^2})\exp(il\varphi)$$
$$\times\frac{1}{\sqrt{2}}\begin{bmatrix}(\cos^2\varphi\cos\theta+\sin^2\varphi)\pm i(\cos\theta-1)\sin\varphi\cos\varphi\\(\cos\theta-1)\sin\varphi\cos\varphi\pm i(\cos^2\varphi+\sin^2\varphi\cos\theta)\\\sin\theta\exp(\pm i\varphi)\end{bmatrix}\mathrm{e}^{ik\cdot z_s\cos\theta}\mathrm{e}^{ik\cdot r_s\sin\theta\cos(\varphi-\varphi_s)}\mathrm{d}\varphi\mathrm{d}\theta\,. \tag{S9}$$

For the Eq. S9, with the mathematical relations

$$\begin{aligned}\int_0^{2\pi}\cos(N\phi)\mathrm{e}^{ix\cos(\phi-\varphi)}\mathrm{d}\phi&=2\pi(i^N)J_N(x)\cos(N\varphi)\\\int_0^{2\pi}\sin(N\phi)\mathrm{e}^{ix\cos(\phi-\varphi)}\mathrm{d}\phi&=2\pi(i^N)J_N(x)\sin(N\varphi)\end{aligned}\,, \tag{S10}$$

the x,y,z polarized components of the electric field at the point (r_s,φ_s,z_s) of the focusing field can be derived as

$$
\begin{aligned}
E_{\pm,x}(r_s,\varphi_s,z_s) &= \frac{-ikf}{2\sqrt{2}}\int_0^{\theta_{\max}} \sin\theta\sqrt{\cos\theta}\,(\frac{\sqrt{2}f\sin\theta}{w_0})^{|l|}\exp(-\frac{f^2(\sin\theta)^2}{w_0^2})\cdot \mathrm{e}^{ik\cdot z_s\cos\theta} \\
&\times\begin{bmatrix}(\cos\theta-1)\cdot(i)^{l\pm2}\cdot J_{l\pm2}(k\cdot r_s\sin\theta)\exp(i(l\pm2)\varphi_s) \\ +(\cos\theta+1)\cdot(i)^{l}\cdot J_{l}(k\cdot r_s\sin\theta)\exp(il\varphi_s)\end{bmatrix}\mathrm{d}\theta \\
E_{\pm,y}(r_s,\varphi_s,z_s) &= \frac{-ikf}{2\sqrt{2}}\int_0^{\theta_{\max}} \sin\theta\sqrt{\cos\theta}\,(\frac{\sqrt{2}f\sin\theta}{w_0})^{|l|}\exp(-\frac{f^2(\sin\theta)^2}{w_0^2})\cdot \mathrm{e}^{ik\cdot z_s\cos\theta} \\
&\times\begin{bmatrix}\frac{(\cos\theta\pm1)}{2}\cdot(i)^{l\pm2}\cdot J_{l\pm2}(k\cdot r_s\sin\theta)\cdot\exp(i(l\pm2)\varphi_s) \\ +(i(\frac{\cos\theta\pm1}{2}))\cdot(i)^{l}\cdot J_{l}(k\cdot r_s\sin\theta)\exp(il\varphi_s)\end{bmatrix}\mathrm{d}\theta \\
E_{\pm,z}(r_s,\varphi_s,z_s) &= \frac{-ikf}{\sqrt{2}}\exp(l\pm1)i\varphi_s\int_0^{\theta_{\max}}(\frac{\sqrt{2}f\sin\theta}{w_0})^{|l|}\exp(-\frac{f^2(\sin\theta)^2}{w_0^2})\sin^2\theta\sqrt{\cos\theta}\cdot\mathrm{e}^{ik\cdot z_s\cos\theta} \\
&\times(i)^{l\pm1}\cdot J_{l\pm1}(k\cdot r_s\sin\theta)\mathrm{d}\theta
\end{aligned}
\quad , \qquad \text{(S11)}
$$

where $J_N(x)$ is an N-order Bessel function of the first kind. The subscripts $''+''$ and $''-''$ indicate that the polarization of the incident beams is RCP and LCP, respectively.

Numerical derivation of focused right-handed circularly polarized Laguerre Gaussian beams based on Debye-Wolf theory

Similar to the manuscript description, we also use Eq. 8 in the main manuscript to numerically calculate the intensity distribution and phase distribution of the focusing field of the right-handed circularly polarized (RCP) vortex beam.

Figure. S1 shows the total intensity distributions of the circular polarization and longitudinal z components of the RCP LG beams ($\sigma=+1$ and $l=\pm1$) in the focal plane, respectively. Also, the corresponding phase distributions of the circular ($+,-$) and longitudinal (z) polarization components in the focal plane are depicted. As shown in Figs. S1A and S1B, we can find that the total intensity profile is donut-shaped when $l=+1$ (total angular momentum $|L|=2$), while there is solid center-shaped when $l=-1$ (total angular momentum $|L|=0$). The calculation shows that such phenomena are mainly contributed by the z-component of the focused fields. Fig. S1A shows the phase distribution of the circular polarized and longitudinal components of the RCP LG beam carrying $l=+1$, and phase changes are 2π, 6π and 4π, respectively, which matches the vortex factor contained in each component of Eq. 5 in the manuscript. Similarly, we can find that the phase change of each component of the RCP LG beam carrying $l=-1$ is -2π, 2π, and 0 (Fig. S1B), respectively, which also corresponds to the vortex factors of each component in Eq. 5 in the manuscript. Therefore, the interaction of the vortex factor carried by the LG beam itself with the helicity-dependent vortex factor produced by the focusing process results in a different longitudinal component of the focusing field, which determines the difference between the dark (with singularity) and bright (without singularity) spots at the vicinity of the beam axis.

Farfield characterization of circularly polarized Laguerre Gaussian beams

Based on the optical system shown in Fig. 3 of the manuscript, the measured farfield intensity distributions of the RCP and LCP LG beams are depicted in Fig. S2. From Fig. S2, we can observe that for beams with the same polarization but different topological charges or with different polarizations but the same topological charge, the farfield intensity distribution exhibits symmetric hollow patterns.

Nearfield characterization of circularly polarized Laguerre Gaussian beams using PiFM

The nearfield optical force distribution of RCP and LCP LG beams with $l = \pm 1$ measured by our experiment is shown in Fig. S3. As shown in Figs. S3A and S3B, the total angular momentum of the RCP LG beam with $l = +1$ and LCP LG beam with $l = -1$ is $|L| = 2$, and the nearfield optical force presents a donut-shaped profile distribution. For the total angular momentum $|L| = 0$ of the RCP LG beam with $l = -1$ and LCP LG beam with $l = +1$, the nearfield optical force presents a bright-center distribution.

The time and azimuthal averages of the photo-induced force based on fitting of the measured force

To map the electric field distribution in the focal plane, we measure the interaction force between the probe tip and its image dipole under various incident light conditions. The longitudinal optical force distributions obtained are depicted in Figs. 4A and 4B and Figs. S4A and S4B. We derive Eq. 16 using the dipole approximation model, which facilitates numerical evaluation of the longitudinal optical force. From Eq. 16, the longitudinal optical force arises from both transverse and longitudinal optical fields at the focal plane. The relative contributions of these fields depend on the effective dipole of the tip and its image dipole. Specifically, the polarizability of the image dipole α_{img} is proportional to the polarizability of the tip α_{tip} and the scale factor is $(\varepsilon_g - \varepsilon_a)/(\varepsilon_g + 2\varepsilon_a)$ (where ε_g and ε_a is the dielectric constants of glass and air) (*68*). To evaluate the respective contributions of transverse and longitudinal optical fields to the measured longitudinal optical force, we utilize the measured force distribution to fit the dependence of this force on the transverse and longitudinal electric fields of the incident light field according to Eq. 16.

In addition, according to Figs. 4A and 4B, and Figs. S4A and S4B, we can find that the optical force maps measured by PiFM have a certain amount of background noise, and the fitting model after considering the background noise is

$$\left\langle \mathbf{F}_{\text{pho,z}} \right\rangle \approx -\frac{3}{8\pi\varepsilon_0 d^4} \operatorname{Re}\left(\alpha_{\text{tip,r}} \alpha_{\text{img,r}} \left| E_r^{focal} \right|^2 - 2\alpha_{\text{tip,z}} \alpha_{\text{img,z}} \left| E_z^{focal} \right|^2 \right) + I_n , \qquad \text{(S12)}$$

where I_n is the background noise of the experimental optical force map. d is the distance between the image and tip dipoles, which is estimated as 5 nm. The transverse and longitudinal light intensities ($\left| E_r^{focal} \right|^2$ and $\left| E_z^{focal} \right|^2$) are calculated by Eq. 8, after which the coefficient ratios of the transverse and longitudinal optical fields are obtained by parametric scanning. To mitigate the effects of anisotropy in the experimentally obtained force diagrams, we plot the relationship between the time- and azimuthally-averaged optical force and the radial distance from the beam axis ($\rho = \sqrt{x^2 + y^2}$). By comparing the experimental and fitted curves, we finalize the coefficient ratios of the transverse and longitudinal optical fields.

Through fitting, for the LCP LG beam with $l = +1$ topological charge and LCP LG beam with $l = -1$ topological charge, the fitted optical force distributions are shown in Fig. 4C and Fig. 4D, and the time- and azimuth-averaged optical force profiles are shown in Fig. 4E and Fig. 4F. Under the conditions of incident light being the RCP LG beam with $l = +1$ topological charge and the RCP LG beam with $l = -1$ topological charge, the fitted optical force distribution are shown in Fig. S4C and Fig. S4D, and the time- and azimuth-averaged optical force profiles are shown in Fig. S4E and Fig. S4F. Accordingly, the corresponding transverse light field distribution and longitudinal light field retrieved are shown in Fig. S5.

<u>Matching of experimental nearfield optical force with numerical fits for right-handed circularly polarized Laguerre Gaussian beams</u>

As shown in Fig. S4, the experimentally obtained nearfield optical force distributions of RCP LG beams carrying $l = \pm 1$ show a donut-shape and a bright-center distribution, respectively. Compared with the CCD images obtained before focusing on the PiFM system (as depicted in Figs. S4G and S4H), the LCP LG beams with equal polarization helicity and different topological charges exhibit a symmetric donut distribution in the farfield. The depicted Figs. S4C and S4D illustrate the optical force distribution obtained using the numerical model. Following this, we numerically fit the radial dependence of the experimental force data with the radial distance to the beam axis as shown in Figs. S4E and S4F. Moreover, the corresponding transverse and longitudinal light field distributions retrieved are shown in Fig. S5C and S5D.

<u>The measured force map with respect to the z-axis near the focal point</u>

The optical force distributions along the z-direction are obtained by scanning the optical field distribution at different focal lengths by a piezoelectric cell controlling the tip of the PiFM system. As shown in Fig. S6A and S6B, we experimentally map the optical forces at the focal plane (defined as $z = 0$) and scan the z-position in 100 nm intervals to $z = +/-300$ nm, for the LCP LG ($l = -1$) and LCP LG ($l = +1$) beams. Then we plot the simulated transverse and longitudinal optical fields from the fitted model in the corresponding case by COMSOL Multiphysics as shown in Fig. S6Cand S6D. The evolution of the measured optical forces fits with the simulated field and shows a clear trend of focusing. In particular, by comparing the optical force distributions with the transverse and longitudinal nearfield intensity distributions of light, one can find that near the focal plane, the total optical forces depend mainly on the longitudinal nearfield component. This feature provides an important merit of the PiFM which is especially sensitive to the longitudinal nearfield.

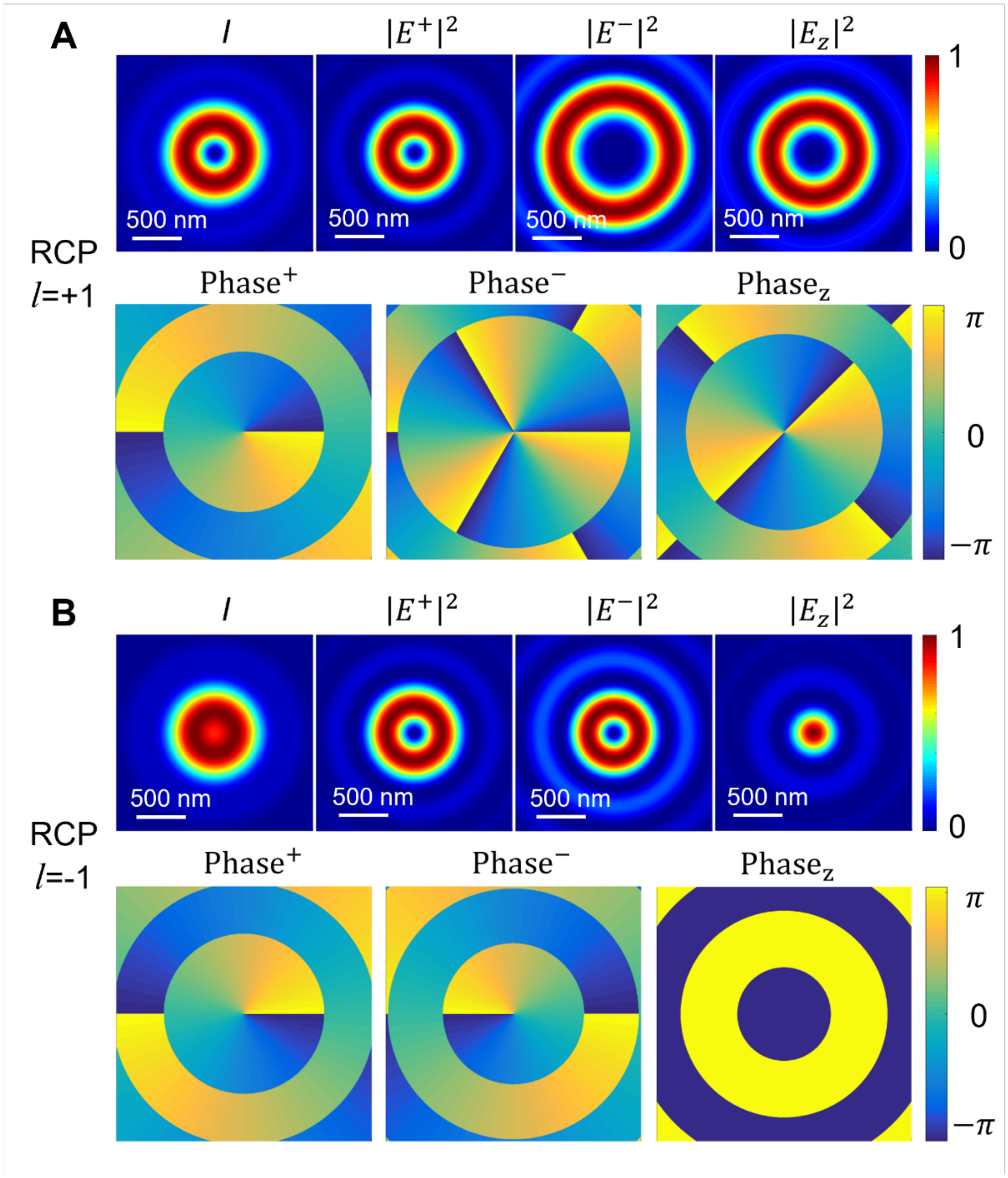

Fig. S1.

Simulation results of tightly focused right circularly polarized vortex beams. (**A**) The total intensity $I = |E_x|^2 + |E_y|^2 + |E_z|^2$ and corresponding circular polarization and longitudinal components $|E^+|^2, |E^-|^2, |E_z|^2$, respectively, and phase distributions of each component $(\text{Phase}^+, \text{Phase}^-, \text{Phase}_z)$ of the RCP LG beam with $l = +1$, respectively; (**B**) The total intensity $I = |E_x|^2 + |E_y|^2 + |E_z|^2$ and corresponding circular polarization and longitudinal components $|E^+|^2, |E^-|^2, |E_z|^2$, respectively, and phase distributions of each component $(\text{Phase}^+, \text{Phase}^-, \text{Phase}_z)$ of the RCP LG beam with $l = -1$, respectively.

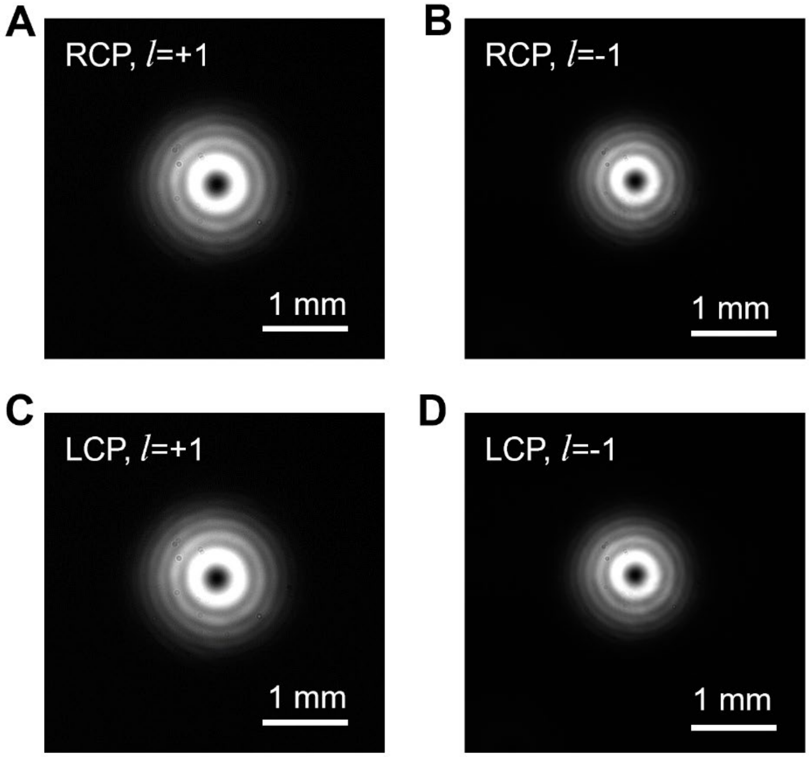

Fig. S2.

Distribution of intensity detected by farfield CCD. (**A**) RCP LG beam with $l=+1$ topological charge; (**B**) RCP LG beam with $l=-1$ topological charge; (**C**) LCP LG beam with $l=+1$ topological charge; (**D**) LCP LG beam with $l=-1$ topological charge.

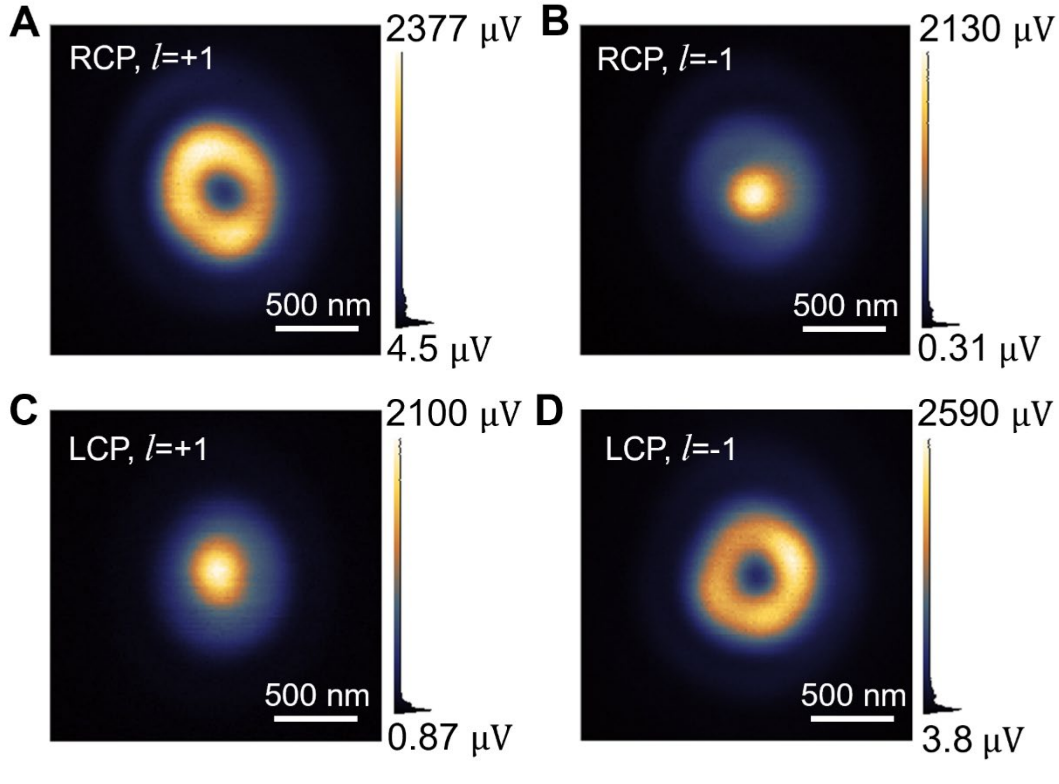

Fig. S3.

Nearfield force distributions measured directly through PiFM. (**A**) RCP LG beam with $l = +1$ topological charge; (**B**) RCP LG beam with $l = -1$ topological charge; (**C**) LCP LG beam with $l = +1$ topological charge; (**D**) LCP LG beam with $l = -1$ topological charge.

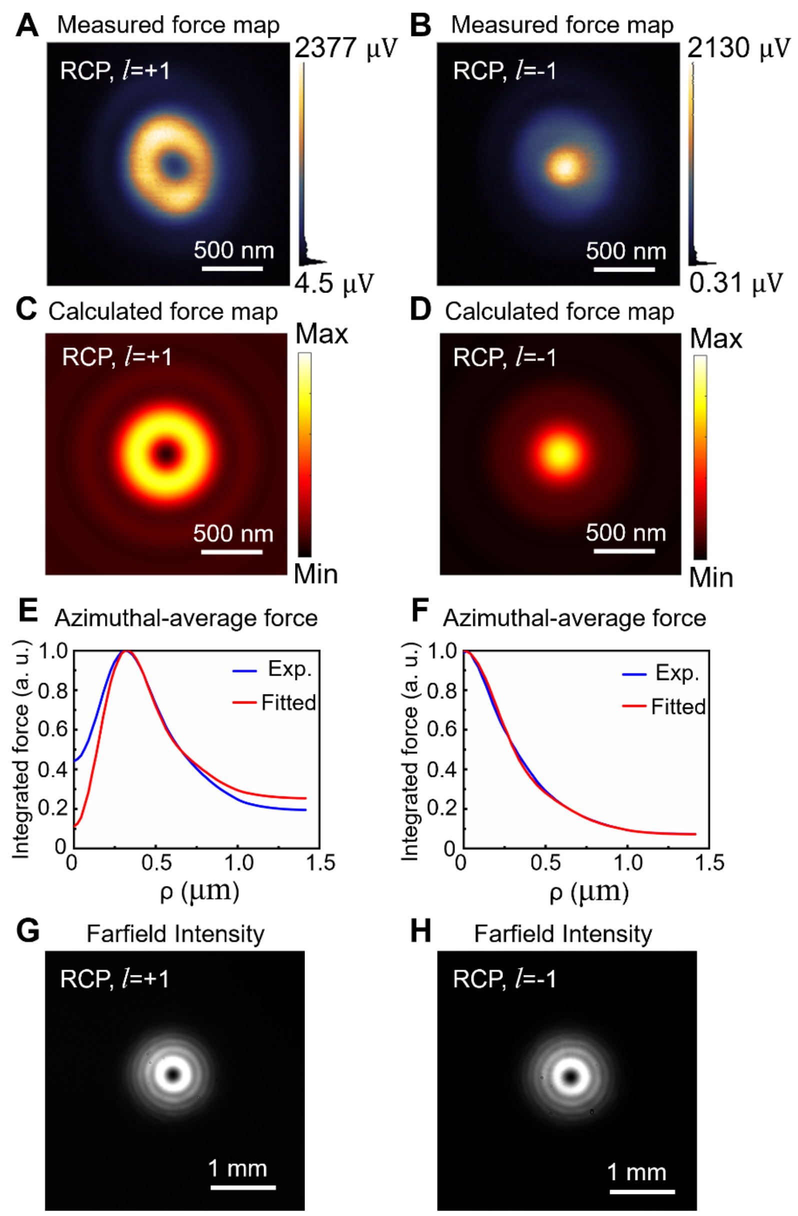

Fig. S4.

Nearfield force and farfield intensity distributions and simulation results of right-handed circularly polarized vortex beams. PiFM direct nearfield force measurement distribution of (**A**) RCP LG beams with $l=+1$ topological charge and (**B**) RCP LG beams with $l=-1$ topological charge; Optical force distribution obtained by numerical model fitting for (**C**) the RCP LG beam with $l=+1$ topological charge and (**D**) the RCP LG beam with $l=-1$ topological charge; Experimental results and fitted numerical model of azimuthal-average force versus radial distance from the beam axis ρ for (**E**) RCP LG beam with $l=+1$ topological charge and (**F**) RCP LG beam with $l=-1$ topological charge. Distributions of intensity detected by farfield CCD for (**G**) RCP LG beam with $l=+1$ topological charge and (**H**) RCP LG beam with $l=-1$ topological charge.

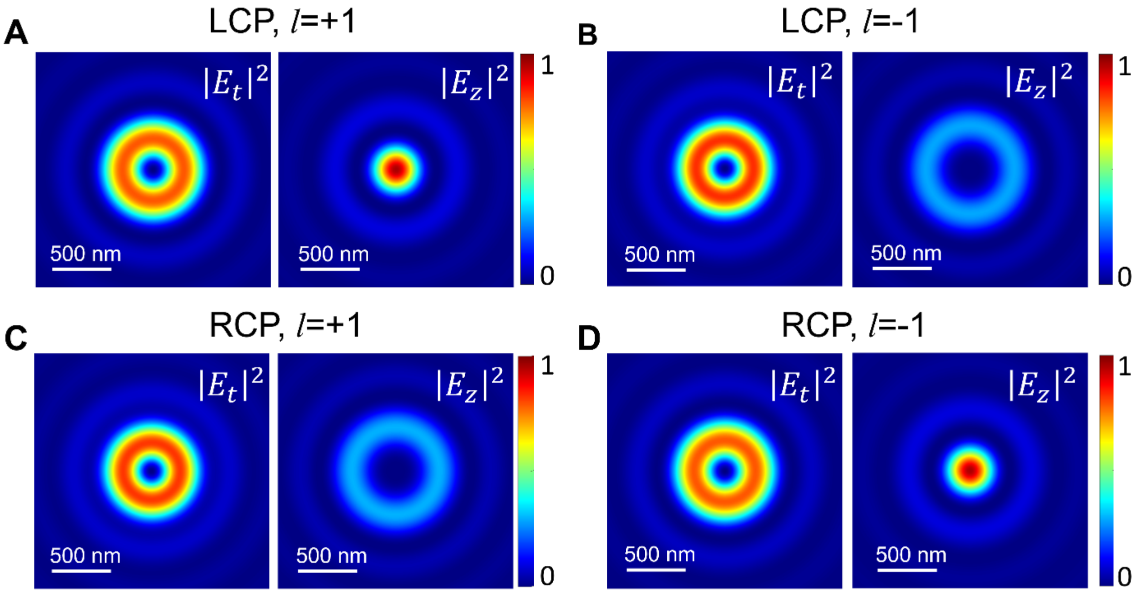

Fig. S5.

Transverse and longitudinal intensity distributions ($|E_t|^2$ and $|E_z|^2$) of tightly focused LCP and RCP vortex beams obtained using the fitted model. (**A**) LCP LG beams with $l=+1$ topological charge; (**B**) LCP LG beams with $l=-1$ topological charge; (**C**) RCP LG beam with $l=+1$ topological charge; (**D**) RCP LG beam with $l=-1$ topological charge.

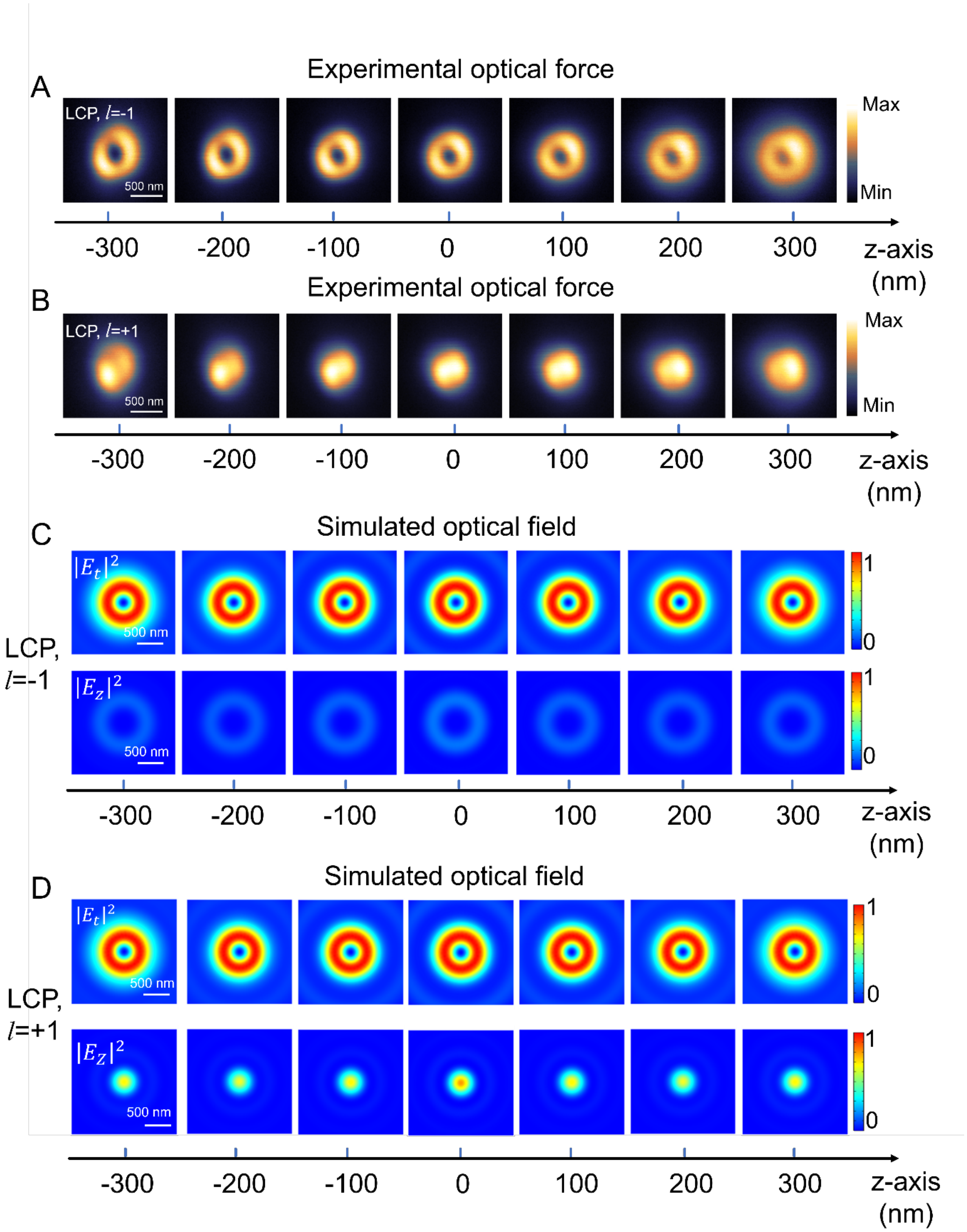

Fig. S6.

The measured force maps and the simulated transverse and longitudinal optical field with respect to the z-axis near the focal point for the LCP LG beams with $l = \pm 1$ topological charge. (**A**) The measured force map of the LCP LG beam with $l = -1$ topological charge; (**B**)

The measured force map of the LCP LG beams with $l = +1$ topological charge; (**C**) The simulated transverse and longitudinal optical field of the LCP LG beam with $l = -1$ topological charge; (**D**) The simulated transverse and longitudinal optical field of the LCP LG beam with $l = +1$ topological charge.

REFERENCES AND NOTES

1. J. H. Poynting, The wave motion of a revolving shaft, and a suggestion as to the angular momentum in a beam of circularly polarised light. *Proc. R. Soc. Lond. A* **82**, 560–567 (1909).

2. L. Allen, M. W. Beijersbergen, R. J. C. Spreeuw, J. P. Woerdman, Orbital angular momentum of light and the transformation of Laguerre-Gaussian laser modes. *Phys. Rev. A* **45**, 8185–8189 (1992).

3. A. M. Yao, M. J. Padgett, Orbital angular momentum: Origins, behavior and applications. *Adv. Opt. Photon.* **3**, 161–204 (2011).

4. V. S. Liberman, B. Y. Zel'dovich, Spin-orbit interaction of a photon in an inhomogeneous medium. *Phys. Rev. A* **46**, 5199–5207 (1992).

5. L. Marrucci, E. Karimi, S. Slussarenko, B. Piccirillo, E. Santamato, E. Nagali, F. Sciarrino, Spin-to-orbital conversion of the angular momentum of light and its classical and quantum applications. *J. Opt.* **13**, 064001 (2011).

6. K. Y. Bliokh, M. A. Alonso, E. A. Ostrovskaya, A. Aiello, Angular momenta and spin-orbit interaction of nonparaxial light in free space. *Phys. Rev. A* **82**, 063825 (2010).

7. K. Y. Bliokh, F. J. Rodríguez-Fortuño, F. Nori, A. V. Zayats, Spin–orbit interactions of light. *Nat. Photon.* **9**, 796–808 (2015).

8. K. Yu. Bliokh, Y. P. Bliokh, Conservation of angular momentum, transverse shift, and spin hall effect in reflection and refraction of an electromagnetic wave packet. *Phys. Rev. Lett.* **96**, 073903 (2006).

9. L. Marrucci, C. Manzo, D. Paparo, Optical spin-to-orbital angular momentum conversion in inhomogeneous anisotropic media. *Phys. Rev. Lett.* **96**, 163905 (2006).

10. X. Ling, X. Zhou, K. Huang, Y. Liu, C.-W. Qiu, H. Luo, S. Wen, Recent advances in the spin Hall effect of light. *Rep. Prog. Phys.* **80**, 066401 (2017).

11. S. Fu, C. Guo, G. Liu, Y. Li, H. Yin, Z. Li, Z. Chen, Spin-orbit optical Hall effect. *Phys. Rev. Lett.* **123**, 243904 (2019).

12. B. Roy, N. Ghosh, A. Banerjee, S. D. Gupta, S. Roy, Manifestations of geometric phase and enhanced spin Hall shifts in an optical trap. *New J. Phys.* **16**, 083037 (2014).

13. O. G. Rodríguez-Herrera, D. Lara, K. Y. Bliokh, E. A. Ostrovskaya, C. Dainty, Optical nanoprobing via spin-orbit interaction of light. *Phys. Rev. Lett.* **104**, 253601 (2010).

14. X. Zhou, Z. Xiao, H. Luo, S. Wen, Experimental observation of the spin Hall effect of light on a nanometal film via weak measurements. *Phys. Rev. A* **85**, 043809 (2012).

15. G. D. M. Jeffries, J. S. Edgar, Y. Zhao, J. P. Shelby, C. Fong, D. T. Chiu, Using polarization-shaped optical vortex traps for single-cell nanosurgery. *Nano Lett.* **7**, 415–420 (2007).

16. Y. Zhao, J. S. Edgar, G. D. M. Jeffries, D. McGloin, D. T. Chiu, Spin-to-orbital angular momentum conversion in a strongly focused optical beam. *Phys. Rev. Lett.* **99**, 073901 (2007).

17. K. Y. Bliokh, E. A. Ostrovskaya, M. A. Alonso, O. G. Rodríguez-Herrera, D. Lara, C. Dainty, Spin-to-orbital angular momentum conversion in focusing, scattering, and imaging systems. *Opt. Express* **19**, 26132–26149 (2011).

18. Y. Liu, Y. Hwang, G. Si, Q. Wang, D. Wang, Spin-to-orbital angular momentum conversion in symmetric dielectric nanorings. *Appl. Phys. Lett.* **118**, 161106 (2021).

19. R. C. Devlin, A. Ambrosio, N. A. Rubin, J. P. B. Mueller, F. Capasso, Arbitrary spin-to–orbital angular momentum conversion of light. *Science* **358**, 896–901 (2017).

20. L. Bosanac, T. Aabo, P. M. Bendix, L. B. Oddershede, Efficient optical trapping and visualization of silver nanoparticles. *Nano Lett.* **8**, 1486–1491 (2008).

21. Y. Zhang, C. Min, X. Dou, X. Wang, H. P. Urbach, M. G. Somekh, X. Yuan, Plasmonic tweezers: For nanoscale optical trapping and beyond. *Light Sci. Appl.* **10**, 59 (2021).

22. Y. Yang, Y. Ren, M. Chen, Y. Arita, C. Rosales-Guzmán, Optical trapping with structured light: A review. *Adv. Photon.* **3**, 034001 (2021).

23. M. Rajaei, M. A. Almajhadi, J. Zeng, H. K. Wickramasinghe, Near-field nanoprobing using Si tip-Au nanoparticle photoinduced force microscopy with 120:1 signal-to-noise ratio, sub-6-nm resolution. *Opt. Express* **26**, 26365–26376 (2018).

24. G. Araneda, S. Walser, Y. Colombe, D. B. Higginbottom, J. Volz, R. Blatt, A. Rauschenbeutel, Wavelength-scale errors in optical localization due to spin–orbit coupling of light. *Nat. Phys.* **15**, 17–21 (2019).

25. Y. Zhang, J. Bai, Improving the recording ability of a near-field optical storage system by higher-order radially polarized beams. *Opt. Express* **17**, 3698–3706 (2009).

26. X. Ouyang, Y. Xu, M. Xian, Z. Feng, L. Zhu, Y. Cao, S. Lan, B.-O. Guan, C.-W. Qiu, M. Gu, X. Li, Synthetic helical dichroism for six-dimensional optical orbital angular momentum multiplexing. *Nat. Photon.* **15**, 901–907 (2021).

27. J. Parker, C. W. Peterson, Y. Yifat, S. A. Rice, Z. Yan, S. K. Gray, N. F. Scherer, Optical matter machines: Angular momentum conversion by collective modes in optically bound nanoparticle arrays. *Optica* **7**, 1341–1348 (2020).

28. V. V. Kotlyar, A. G. Nalimov, A. A. Kovalev, A. P. Porfirev, S. S. Stafeev, Spin-orbit and orbit-spin conversion in the sharp focus of laser light: Theory and experiment. *Phys. Rev. A* **102**, 033502 (2020).

29. I. Rajapaksa, K. Uenal, H. K. Wickramasinghe, Image force microscopy of molecular resonance: A microscope principle. *Appl. Phys. Lett.* **97**, 073121 (2010).

30. A. A. Sifat, J. Jahng, E. O. Potma, Photo-induced force microscopy (PiFM) – Principles and implementations. *Chem. Soc. Rev.* **51**, 4208–4222 (2022).

31. M. Poblet, Y. Li, E. Cortés, S. A. Maier, G. Grinblat, A. V. Bragas, Direct detection of optical forces of magnetic nature in dielectric nanoantennas. *Nano Lett.* **20**, 7627–7634 (2020).

32. J. Zeng, M. Darvishzadeh-Varcheie, M. Albooyeh, M. Rajaei, M. Kamandi, M. Veysi, E. O. Potma, F. Capolino, H. K. Wickramasinghe, Exclusive magnetic excitation enabled by structured light illumination in a nanoscale mie resonator. *ACS Nano* **12**, 12159–12168 (2018).

33. L. M. Otter, M. W. Förster, E. Belousova, P. O'Reilly, D. Nowak, S. Park, S. Clark, S. F. Foley, D. E. Jacob, Nanoscale chemical imaging by photo-induced force microscopy: Technical aspects and application to the geosciences. *Geostand. Geoanal. Res.* **45**, 5–27 (2021).

34. J. Yamanishi, H. Yamane, Y. Naitoh, Y. J. Li, N. Yokoshi, T. Kameyama, S. Koyama, T. Torimoto, H. Ishihara, Y. Sugawara, Optical force mapping at the single-nanometre scale. *Nat. Commun.* **12**, 3865 (2021).

35. J. Zeng, F. Huang, C. Guclu, M. Veysi, M. Albooyeh, H. K. Wickramasinghe, F. Capolino, Sharply focused azimuthally polarized beams with magnetic dominance: Near-field characterization at nanoscale by photoinduced force microscopy. *ACS Photonics* **5**, 390–397 (2018).

36. J. Zeng, M. Albooyeh, M. Rajaei, A. A. Sifat, E. O. Potma, H. K. Wickramasinghe, F. Capolino, Direct detection of photoinduced magnetic force at the nanoscale reveals magnetic nearfield of structured light. *Sci. Adv.* **8**, eadd0233 (2022).

37. J. Jahng, J. G. Son, H. Kim, J. Park, T. G. Lee, E. S. Lee, Direct chemical imaging of ligand-functionalized single nanoparticles by photoinduced force microscopy. *J. Phys. Chem. Lett.* **11**, 5785–5791 (2020).

38. D. Nowak, W. Morrison, H. K. Wickramasinghe, J. Jahng, E. Potma, L. Wan, R. Ruiz, T. R. Albrecht, K. Schmidt, J. Frommer, D. P. Sanders, S. Park, Nanoscale chemical imaging by photoinduced force microscopy. *Sci. Adv.* **2**, e1501571 (2016).

39. R. A. Murdick, W. Morrison, D. Nowak, T. R. Albrecht, J. Jahng, S. Park, Photoinduced force microscopy: A technique for hyperspectral nanochemical mapping. *Jpn. J. Appl. Phys.* **56**, 08LA04 (2017).

40. J. Li, J. Pang, Z.-D. Yan, J. Jahng, J. Li, W. Morrison, J. Liang, Q.-Y. Zhang, X.-H. Xia, Antenna enhanced infrared photoinduced force imaging in aqueous environment with super-resolution and hypersensitivity. *CCS Chem.* **4**, 2738–2747 (2022).

41. J. Jahng, D. A. Fishman, S. Park, D. B. Nowak, W. A. Morrison, H. K. Wickramasinghe, E. O. Potma, Linear and nonlinear optical spectroscopy at the nanoscale with photoinduced force microscopy. *Acc. Chem. Res.* **48**, 2671–2679 (2015).

42. I. Rajapaksa, H. K. Wickramasinghe, Raman spectroscopy and microscopy based on mechanical force detection. *Appl. Phys. Lett.* **99**, 161103 (2011).

43. J. Yamanishi, H.-Y. Ahn, H. Okamoto, Nanoscopic observation of chiro-optical force. *Nano Lett.* **23**, 9347–9352 (2023).

44. M. Kamandi, M. Albooyeh, M. Veysi, M. Rajaei, J. Zeng, H. K. Wickramasinghe, F. Capolino, Unscrambling structured chirality with structured light at the nanoscale using photoinduced force. *ACS Photonics* **5**, 4360–4370 (2018).

45. S. Kwon, J. M. Kim, P. J. Ma, W. Guan, S. Nam, Near-field nano-optical imaging of van der Waals materials. *Adv. Phys. Res.* **2**, 2300009 (2023).

46. T. U. Tumkur, M. A. Hurier, M. D. Pichois, M. Vomir, B. Donnio, J. L. Gallani, M. V. Rastei, Photoinduced atomic force spectroscopy and imaging of two-dimensional materials. *Phys. Rev. Appl.* **11**, 044066 (2019).

47. B. Ji, A. Kenaan, S. Gao, J. Cheng, D. Cui, H. Yang, J. Wang, J. Song, Label-free detection of biotoxins *via* a photo-induced force infrared spectrum at the single-molecular level. *Analyst* **144**, 6108–6117 (2019).

48. J. Li, J. Jahng, J. Pang, W. Morrison, J. Li, E. S. Lee, J.-J. Xu, H.-Y. Chen, X.-H. Xia, Tip-enhanced infrared imaging with sub-10 nm resolution and hypersensitivity. *J. Phys. Chem. Lett.* **11**, 1697–1701 (2020).

49. Y. Bai, M. Dong, M. Zhang, Y. Yang, Properties of a tightly focused circularly polarized anomalous vortex beam and its optical forces on trapped nanoparticles. *Nanoscale Res. Lett.* **14**, 252 (2019).

50. B. Richards, E. Wolf, D. Gabor, Electromagnetic diffraction in optical systems, II. Structure of the image field in an aplanatic system. *Proc. R. Soc. Lond. A* **253**, 358–379 (1959).

51. Y. Hu, Z. Wang, X. Wang, S. Ji, C. Zhang, J. Li, W. Zhu, D. Wu, J. Chu, Efficient full-path optical calculation of scalar and vector diffraction using the Bluestein method. *Light Sci. Appl.* **9**, 119 (2020).

52. Q. Zhan, Cylindrical vector beams: From mathematical concepts to applications. *Adv. Opt. Photon.* **1**, 1–57 (2009).

53. P. B. Monteiro, P. A. M. Neto, H. M. Nussenzveig, Angular momentum of focused beams: Beyond the paraxial approximation. *Phys. Rev. A* **79**, 033830 (2009).

54. F. Huang, V. Ananth Tamma, Z. Mardy, J. Burdett, H. K. Wickramasinghe, Imaging nanoscale electromagnetic near-field distributions using optical forces. *Sci. Rep.* **5**, 10610 (2015).

55. J. D. Jackson, *Classical Electrodynamics* (John Wiley & Sons, 2021).

56. P. Grahn, A. Shevchenko, M. Kaivola, Electromagnetic multipole theory for optical nanomaterials. *New J. Phys.* **14**, 093033 (2012).

57. S. S. Kruk, R. Camacho-Morales, L. Xu, M. Rahmani, D. A. Smirnova, L. Wang, H. H. Tan, C. Jagadish, D. N. Neshev, Y. S. Kivshar, Nonlinear optical magnetism revealed by second-harmonic generation in nanoantennas. *Nano Lett.* **17**, 3914–3918 (2017).

58. X. Xu, M. Nieto-Vesperinas, Y. Zhou, Y. Zhang, M. Li, F. J. Rodríguez-Fortuño, S. Yan, B. Yao, Gradient and curl optical torques. *Nat. Commun.* **15**, 6230 (2024).

59. Y. Zhou, X. Xu, Y. Zhang, M. Li, S. Yan, M. Nieto-Vesperinas, B. Li, C.-W. Qiu, B. Yao, Observation of high-order imaginary Poynting momentum optomechanics in structured light. *Proc. Natl. Acad. Sci. U.S.A.* **119**, e2209721119 (2022).

60. F. Nan, F. J. Rodríguez-Fortuño, S. Yan, J. J. Kingsley-Smith, J. Ng, B. Yao, Z. Yan, X. Xu, Creating tunable lateral optical forces through multipolar interplay in single nanowires. *Nat. Commun.* **14**, 6361 (2023).

61. Z. Zhang, H. Zhao, S. Wu, T. Wu, X. Qiao, Z. Gao, R. Agarwal, S. Longhi, N. M. Litchinitser, L. Ge, L. Feng, Spin–orbit microlaser emitting in a four-dimensional Hilbert space. *Nature* **612**, 246–251 (2022).

62. M. Veysi, C. Guclu, F. Capolino, Focused azimuthally polarized vector beam and spatial magnetic resolution below the diffraction limit. *J. Opt. Soc. Am. B* **33**, 2265–2277 (2016).

63. A. David, B. Gjonaj, G. Bartal, Two-dimensional optical nanovortices at visible light. *Phys. Rev. B* **93**, 121302 (2016).

64. Y. Chen, J. Chu, X. Xu, Plasmonic multibowtie aperture antenna with fano resonance for nanoscale spectral sorting. *ACS Photonics* **3**, 1689–1697 (2016).

65. Y. Zhao, A. A. E. Saleh, M. A. van de Haar, B. Baum, J. A. Briggs, A. Lay, O. A. Reyes-Becerra, J. A. Dionne, Nanoscopic control and quantification of enantioselective optical forces. *Nat. Nanotech* **12**, 1055–1059 (2017).

66. A. A. Sifat, F. Capolino, E. O. Potma, Force detection of electromagnetic chirality of tightly focused laser beams. *ACS Photonics* **9**, 2660–2667 (2022).

67. K. A. Forbes, D. L. Andrews, "Spin-orbit coupling in vortex light: Can it be revealed in fundamental electronic transitions?" *Proc. SPIE.* **10732**, 108–117 (2018).

68. L. Novotny, B. Hecht, *Principles of Nano-Optics* (Cambridge Univ. Press, 2012).
